# An integrated approach for designing in-time and economically sustainable emergency care networks: A case study in the public sector

**DOI:** 10.1371/journal.pone.0234984

**Published:** 2020-06-22

**Authors:** Miguel Ortiz-Barrios, Juan-José Alfaro-Saiz

**Affiliations:** 1 Department of Industrial Management, Agroindustry and Operations, Universidad de la Costa CUC, Barranquilla, Colombia; 2 Research Centre on Production Management and Engineering, Universitat Politècnica de València, Valencia, Spain; Sunway University, MALAYSIA

## Abstract

Emergency Care Networks (ECNs) were created as a response to the increased demand for emergency services and the ever-increasing waiting times experienced by patients in emergency rooms. In this sense, ECNs are called to provide a rapid diagnosis and early intervention so that poor patient outcomes, patient dissatisfaction, and cost overruns can be avoided. Nevertheless, ECNs, as nodal systems, are often inefficient due to the lack of coordination between emergency departments (EDs) and the presence of non-value added activities within each ED. This situation is even more complex in the public healthcare sector of low-income countries where emergency care is provided under constraint resources and limited innovation. Notwithstanding the tremendous efforts made by healthcare clusters and government agencies to tackle this problem, most of ECNs do not yet provide nimble and efficient care to patients. Additionally, little progress has been evidenced regarding the creation of methodological approaches that assist policymakers in solving this problem. In an attempt to address these shortcomings, this paper presents a three-phase methodology based on Discrete-event simulation, payment collateral models, and lean six sigma to support the design of in-time and economically sustainable ECNs. The proposed approach is validated in a public ECN consisting of 2 hospitals and 8 POCs (Point of Care). The results of this study evidenced that the average waiting time in an ECN can be substantially diminished by optimizing the cooperation flows between EDs.

## Introduction

Emergency Care Networks (ECNs) are considered complex healthcare systems oriented towards delivering effective emergency services to patients in the most suitable and convenient setting through the alignment of a range of EDs with a patient-centered approach. In fact, the creation of ED networks was suggested by different healthcare institutions in response to the increasing waiting time in emergency units and has been therefore included in various government agendas [1]. Although the integration of EDs has the potential to improve the timely provision of emergency care, several drawbacks have become glaring in both the ED transferring patients and the ED receiving patients which results in non-optimal patient outcomes, long waiting times, and high operational costs. This problem is even more critical considering that the demands of emergency services continue to rise in the future [2]. In the last 20 years, the number of ED admissions increased by 50% in the US [3] whilst, in Australia, the annual admission rate rose by 3.4% (2,017–2,018) [4]. Additionally, in the UK, the number of emergency visits has grown by 42% (1,997–2,017) [5] while this indicator was up to 10% in New Zealand and 5% in Belgium [6]. This problem is more sharpener in developing countries. For instance, in Mexico, hospitals experienced an increased demand of 62% in the last three years [7] while, in Colombia, the Ministry of Health and Social Protection reported that, the number of admissions augmented by 125% from 2,011 to 2,018 [8]. These facts evidence the urgent need for ECNs providing timely diagnosis and care to patients with critical conditions. Although several efforts have been made to address this particular concern, there is still a lack of unified coordination and process inefficiencies across the ECNs.

A critical aspect to be considered in this discussion is the performance of each ED. EDs with serious deficiencies such as overcrowding [9–10], prolonged waiting time, extended length of stay, and high number of patients who leave without seen, may reduce the effectiveness of ECNs in terms of timeliness. Nevertheless, it is not only essential to look into the functioning of emergency departments (EDs) individually but the existing interconnections that regulate the transfer and referral of patients. In this respect, legal, technological, and administrative factors have been found as some of the barriers to the effective functioning of these networks [11]. Operationally, it has been identified that patient needs do not usually correctly match with the ECN capability. It is thus necessary to create robust methodological frameworks that underpin ECN design, planning, and development. Thereby, we can best integrate the EDs into a comprehensive collaboration scheme that ensures the delivery of high-quality emergency care.

Another aspect of concern is the economic gain of each ED within the network. EDs usually refuse to collaborate since they perceive that certain market share may be lost when partnering [12]. In addition, ECNs must be financially viable and sustainable to guarantee the continuous and prompt provision of emergency care over time. In spite of the importance of this aspect, little attention has been paid and is then required to create schemes that ensure equitable and efficient allocation of payments. In some applications, such schemes have been related to operational performance models [13–14]. These models do not only provide support for the utility distribution but generate sufficient information to detect service inefficiencies. With these insights, ECN managers may create cost-effective strategies for improving the delivery of emergency care and the ensuing patient outcomes across the ECN [15].

To address the above-mentioned shortcomings, this paper aims to develop an integrated framework based on Discrete-event simulation, lean manufacturing and six sigma techniques for designing in-time ECNs. Such a framework also includes the creation of a scheme that guarantees the efficient distribution of payments among the ECN participants (EDs). For validation, a public ECN consisting of 2 hospitals and 8 POCs (Point of Care) is considered.

The remainder of this paper is organized as follows. In the second section, approaches used for the design of ECNs are reviewed whereas the proposed methodology for improving the timeliness of these networks is explained in the third section. In the next chapter, a case study of a public ECN is presented to validate the approach here described. Then, the results and analysis are shown in the fifth section. Finally, conclusions and future work are depicted.

### Emergency care networks: Related studies

The effective design and implementation of in-time ECNs have been projected as pillars for addressing the growing demand for emergency services in the future. For a comprehensive analysis of this topic, a review of the most recent reported literature was undertaken by consulting Scopus and Web of Science databases. Specifically, we used two search codes: *“Emergency care network”*, *“Emergency department network”* After careful examination and filtering, only 19 documents (12 articles, 3 reviews, 2 conference papers, and 2 reports) were found from 2,003 (the date on which the first document appeared) to May– 2,019 (search date). Some studies recognized the need for designing ECNs for improving the timeliness of emergency care. For example, Calvello *et al*. [16] suggested creating regionalized, coordinated, and accountable ECNs to address the overcrowding phenomenon. This is consistent with the recommendations provided by Konder and O’Dwyer [17] who determined that collaboration practices may tackle the great patient dissatisfaction with emergency care. In addition, Qayyum and Wardrope [18] concluded that ECNs are necessary to face the increasing demand for emergency and critical care, a problem that has been forecasted in different healthcare systems around the world.

The creation of ECNs, however, must overcome different barriers as identified by Glickman *et al*. [15] who detected large gaps in the evidence base on how ECNs can be organized, coordinated, and measured. In particular, the authors determined that poor linkage of data systems across the EDs and lack of performance measurement models are the main barriers for effective ECN design and implementation. On the other hand, Stoner *et al*. [19] established non-clinical research priorities categorized under the areas of network governance, knowledge translation, and information technology based on the weaknesses detected in pediatric ECNs. Uchimura, da Silva, and Viana [20] found political and governance aspects affecting the effectiveness of ECNs in Brazil. Similar work was undertaken by Konder and O’Dwyer who established that managerial fragmentation was one of the main factors for low integration among EDs in Rio de Janeiro, Brazil [17]. The detection of governance problems within ECNs is also coherent with Qayyum and Wardrope [18] and Almeida *et al*. [21] who expressed that it was necessary to deploy strong leadership and organization considering the need for better coordination and management that ECNs require.

In spite of the research agenda created by the aforementioned studies in relation to ECN functioning, very few studies have aimed to create methodological approaches that guide policymakers towards the effective design and implementation of in-time ECNs in the wild. For instance, Navein and Mcneill [22] described the Surrey Emergency Care System program, an attempt for the development of future integrated and unscheduled ECNs in the UK. However, their approach does not contemplate the individual diagnosis and intervention of the participant EDs before the collaborative scenario. In addition, the initiative does not consider the balance between the demand and ECN capacity, a cornerstone for the correct functioning of these networks in the real world. Harrop proposed an objective data model that can operate at different levels within the network [23]. This framework, however, does not consider interventions in each participant ED before the collaboration, governance arrangements, identification of risks, and creation of payment schemes. Another study was presented by Martínez who provided a conceptual framework for assigning and regionalizing emergency services within an ECN [24]. Nonetheless, it does not establish how this framework can be operationalized in real scenarios. On a different tack, facility-certification models have been proposed for supporting the creation of ECNs. Such traditional models, however, are incapable to balance their capacity with the demand changes [15]. More recently, Gul and Guneri [25], Gul and Guneri [26], and Gul, Guneri, and Gunal [27] have combined discrete-event simulation (DES) with different approaches such as Design of Experiments and Artificial Neural Network (ANN) to model and evaluate the response of an ECN (consisting of five EDs) located in Istanbul when facing increased demand caused by an earthquake. Despite the tremendous effort exposed in these works, several limitations still remain. For instance, the studies focused on designing collaborative scenarios for a particular disaster event. Additionally, they present the same restrictions identified in [23]. It is then evident that there is not an integrated methodology that leads policymakers towards the design and implementation of in-time ECNs considering the entire context of emergency care and collaboration schemes [11, 28].

A starting point for the design and implementation of ECNs may include an individual intervention of the participant EDs to remove the non-value added activities that cause extended waiting times in the emergency rooms. Lean Six Sigma (LSS) is a method that can properly contribute to this particular aim. In fact, the use of LSS has recently gained prominence within EDs. Indeed, Isfahani, Tourani, and Seyedin [29], Habidin, Yahya, and Ramli [30], and Ahmed, Manah, and Islam [31] reviewed the literature related to LSS applications in EDs and concluded that this method has significantly helped ED managers to reduce costs and prevent wastes of time. Specifically, Furterer reported significant reductions in waiting times as well as increased patient satisfaction in an ED after a 3-month project [32]. Another example is provided by Owad, Karim, and Ma who detailed an LSS application in the ED of Asseer Central Hospital in Saudi Arabia where waiting time during patient treatment and other key indicators were also upgraded [33]. In spite of the significant results derived from LSS applications in EDs, there are no studies evidencing its use in ECNs. In fact, LSS may help to slacken the complexity of network interactions so that the number of patient transfers among participant EDs can be optimized.

Another key aspect that should be addressed is the correct functioning of ECNs in the wild. This begins with a design that must be simulated several times to evaluate patient flow, interactions among EDs, and other factors that may worsen waiting times in real scenarios. Given that trials and errors are non-viable, costly, and difficult to implement in the emergency care context, even in large-size ECNs; Discrete-event Simulation (DES) appears to be a suitable method for pretesting the performance of a recently designed ECN. In fact, the use of DES has become popular in the ED context [34]. For instance, Al-Assadi and Hasson utilized DES to maximize patients’ throughput, minimize waiting times and optimize resources in Hilla ED [35]. A similar study was undertaken by Ibrahim *et al*. who developed a computer simulation model in Arena software to test the response of an ED when facing increased levels of demand [36]. Another work is presented by Nuñez-Perez *et al*. who applied DES to model an Accident & Emergency department [37]. In this study, the authors pretested three improvement scenarios to determine the most cost-effective strategy for decreasing patient waiting times. The use of DES for the evaluation of alternative scenarios was also evidenced in Bedoya-Valencia and Kirac [38]. More recently, DES has been combined with different approaches such as Design of Experiments [39], Machine learning [40], ARIMA [41], Six sigma [42–43], and Data Envelopment Analysis [44] to provide more robust results and cover aspects that have not been considered in previous studies (e.g. identification of significant factors, demand forecasting, optimization of resources, etc.). Despite the high number of papers evidencing the application of DES in emergency care processes, studies directly concentrating on ECN design with the use of simulation approaches are largely limited and only focused on disaster events.

An additional issue of importance upon designing in-time ECNs is the definition of an equitable and efficient payment scheme. Such schemes may differ from one country to another whereas they are influenced by the payment and compensation clauses established by each government. In these clauses, some criteria such as, the maximum number of patients that can be seen in each ED and patient type are considered for regulating the collaboration practices. The attempts regarding the creation of payment models for healthcare networks can be found in Barrios, Caballero, and Sánchez [14] and Ortíz-Barrios, Escorcia-Caballero, and Sánchez-Sánchez [45]. These studies implemented a modified version of the collateral payment model for regulating the utility distribution within an integrated network in outpatient internal medicine. In particular, the model considered the correlation between the lead time during the collaboration and the number of patients that a particular hospital received. As a result, the hospital with increased lead time (caused by the collaboration) was economically compensated in accordance with the payment table initially agreed under the collaboration scheme. As observed, the number of studies dealing with payment schemes within healthcare networks is recent and largely limited. Besides, none of the ECN-related studies focused on developing profit distribution agreements that regulate the allocation of payments among participant EDs. The lack of such agreements currently represents a serious limitation for more widespread implementation of ECNs and then becomes a research challenge that should be properly addressed by the practitioners and financial managers involved in this field.

In light of the reported literature, the evidence base on methodologies for creating in-time ECNs is scant and poorly developed with only uncontrolled descriptive studies. Under this consideration, the research question is: ¿How to effectively design in-time and economically sustainable ECNs? To address this gaping hole, this paper presents a three-phase methodology based on DES, LSS, and collateral payment models which overcomes the limitations identified through the literature review. Consequently, the main contribution of this study will be three-fold: i) an integrated approach that helps healthcare managers to design ECNs that timely respond to the growing demand on emergency services, ii) a payment model that grants the efficient and equitable allocation of profits within the ECNs, and iii) the use of LSS and DES for propelling the timely functioning of ECNs.

## The proposed methodology

**Fig 1:**
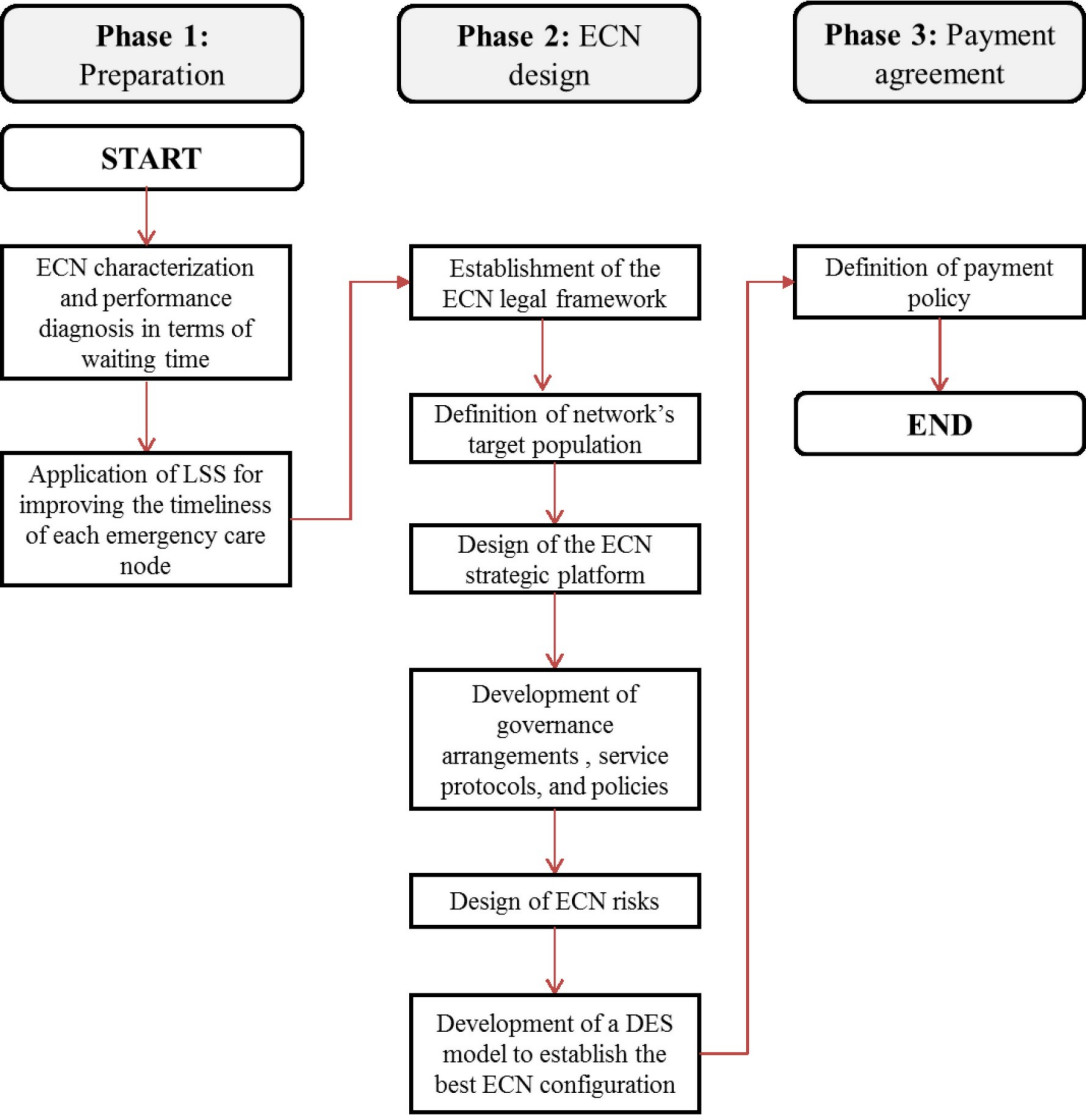
The proposed three-phase methodology for the design of in-time and economically sustainable ECNs.

A three-phase methodology (Fig 1) was proposed to design in-time and economically sustainable ECNs. The description of the steps contained in each phase is shown below.

### Phase 1: Preparation

#### Step 1. ECN characterization and performance diagnosis in terms of waiting time

ECNs can be considered as a nodal scheme where hospitals and POCs are nodes with multiple cooperation flows. Each node should be firstly described and diagnosed to: i) establish the current waiting time that patients may experience when arriving to the emergency room, ii) calculate the installed capacity, iii) identify the type of emergency that can be served in each node, iv) determine the geographical distance between nodes, v) pinpoint the health insurance companies whose patients are enabled to be diagnosed and treated in each node, and vi) estimate the standard deviation and average number of patients that EDs usually receive. Thereby, action plans can be effectively deployed to prepare hospitals and POCs for providing in-time attention within the ECN context.

#### Step 2. Application of LSS for improving the timeliness of each emergency care node

Removing non-value added activities in each emergency care node is critical for diminishing the expected waiting time that patients may experience within a particular ECN. Thereby, an individual preparation of EDs then contributes to the overall timeliness of ECNs and reduces operational drawbacks that may occur when implemented in the wild. LSS, as stated in the literature, can deal with this challenge [46]. The LSS procedure is supported by the DMAIC (Define, Measure, Analyze, Improve, and Control) cycle which is described below [47]:

➢ *Define*: In this point, the waiting time problem is defined based on the estimations provided by Step 1. Also, the project scope, aims, and schedule are detailed through a project charter. Lately, the emergency care processes and stakeholders are fully characterized using SIPOC (Supplier-Input-Process-Output-Customers) diagrams.
➢ *Measure*: The measurement system is assessed to verify whether it provides reliable waiting time data. If this system is proved to be satisfactory, a capability analyze can be then undertaken to determine if the emergency care process meets with the standard waiting time.
➢ *Analyze*: It is necessary to analyze the value chain of emergency care process to identify the variation factors that contribute to the gap between the current waiting time and the desired standard. Some techniques like cause-and-effect analysis, design of experiments, 5 Whys, and Pareto diagram can be applied for this purpose.
➢ *Improve*: Solutions addressing the variation factors, as those supported by lean manufacturing techniques, need to be proposed, prioritized, and implemented by decision makers. The results are then evaluated through a before-and-after study which allows managers to determine whether the timeliness of the emergency care process is closer to the standard.
➢ *Control*: Lately, a control plan including individual X-R charts, is designed to monitor the waiting time behavior and maintaining the improvements achieved through the LSS intervention.

### Phase 2: ECN design

#### Step 1. Establishment of the ECN legal framework

The design of ECNs must be coherent with the regulations established by each government concerning the provision of emergency care services. Therefore, in this step, the decision makers must collect the related laws, agreements, and regulations as well as identify the operational conditions that must be fulfilled before the ECN start. Besides, the current healthcare system should be graphically characterized for ensuring a correct ECN implementation in the wild.

#### Step 2. Definition of network’s target population

Identifying the network’s target population is critical for calculating the demand that can be expected to be covered by the ECN. This is defined through the arrangements concluded between the nodes and the health insurance companies which provide the number of affiliated patients to be potentially admitted within the ECN. Patients who are not covered by the social security should be also considered since, according to international agreements, “patient dumping” is not anymore allowed.

#### Step 3. Design of the ECN strategic platform

In this step, the mission, vision, and strategic goals of ECN are initially defined considering the network’s target population, ECN legal framework, and current performance of participant EDs. After this, ECN corporative values are established taking into account its competitive characteristics, the most important stakeholders’ expectations, critical-to-satisfaction (CTS) factors, and the external conditions. In particular, the stakeholders’ needs regarding the ECN functioning are identified by performing a Voice of Customer (VOC) analysis [48]. The needs with the highest relative frequency are then categorized as CTS factors and should be therefore prioritized by decision-makers when establishing the ECN configuration.

#### Step 4. Development of governance arrangements, service protocols, and policies

As ECNs are integrated by several collaborating EDs, agreed governance structures are necessary for regulating operational functioning and payment flows. Such structure should be led by an ECN Steering Committee (composed by the stakeholders: participant EDs, government, patients, ambulance service companies, and health insurers) whose primary aim is to drive the correct design, implementation, and monitoring of ECNs. In this group, cross-functional communication procedures as well as roles, authority, and responsibilities of each member should be clearly set for ensuring the correct deployment of the predefined strategic platform.

Aside from the governance aspects, service protocols and policies related to the provision of emergency care must be properly defined and disseminated among the emergency units to avoid errors that may endanger patients’ safety. ECN managers should therefore: i) Collect, examine, and select the pertinent guidelines issued by the government and the related regulatory bodies; ii) Classify the selected guidelines into “indoors” and “outdoors” categories. “Indoors” represents the protocols and policies that must be applied within each ED; on the other hand, “Outdoors” refers to those implemented during patient transfers; iii) Identify the domains (Infrastructure—I, medical equipment—ME, procedures and protocols—PP, supporting processes—SP, human resources—HR, supplies/medicines and accessories -SMA, quality—Q, ambulance service—AS, and patient safety—PS) that are related to each guideline; and iv) Disseminate the selected guidelines to the participant EDs before ECN start.

#### Step 5. Definition of ECN risks

Every risk must be adequately managed for avoiding potential failures during ECN functioning [49]. In this sense, risks (i.e. undertriage, patient transfer delay, etc.) must be first identified, evaluated, and prioritized. To do these, it is necessary to establish the process variables of the emergency care service (i.e. waiting time for triage consultation and average length of stay) that are critical for fulfilling the most popular stakeholder expectations. The criticality of these variables is defined by building a matrix specifying how each variable influences each expectation (influence scale– 0: No influence; 1: Extremely weak influence; 2: Weak influence; 3: Moderate influence; 4: Strong influence; 5: Extremely strong influence). Following this, potential failure modes of these variables (in this case, the ECN risks) need to be identified considering the expertise of several emergency care administrators, the pertinent scientific literature, the associated legal framework, and the ECN governance structure. Finally, FMEA (Failure Mode and Effect Analysis) is applied for their assessment and prioritization. Finally, strategies are created to diminish or eliminate the high-risk events if occurred.

#### Step 6. Development of a DES model to establish the ECN configuration

The use of Discrete-event Simulation (DES) in this context is supported by the following arguments: i) recording individual patient waiting time within the ECN is useful, ii) we are searching for cost-effective ECNs considering restricted resources (i.e. number of doctors, number of nurses, etc.), iii) we are interested in analyzing and optimizing the collaboration flows between EDs, iv) DES facilitates engagement with ECN managers through the animation of interactions and resources v) time-to-event behavior is better represented stochastically rather than with time intervals. The application of DES is widely recommended in all these cases according to Karnon *et al*. [50] and Gillespie *et al*. [51]. The DES procedure for effectively establishing the best ECN configuration is as follows:

i) *Input data analysis*: The data collected in Phase 1 is initially prepared through an input analysis. First, an intra-variable independence test is performed to determine whether a specific process variable can be modeled through a statistical distribution function. Assuming that the randomness hypothesis is accepted, a heterogeneity analysis is undertaken using Kruskal-Wallis to classify the data. If the data are homogeneous, one probability distribution is enough to represent data; otherwise, a statistical expression must be defined per each group of data. The goodness-of-fit is validated through a Chi-squared test which also helps to determine the parameters that must be later incorporated into the DES model.

ii) *Creation and validation of a DES model*: The results derived from *Phase 1*, *Step 1* and input data analysis are entered into the simulation software to create a virtual version of the network. The model is then assessed for ensuring its reliability before implementation in the real scenario. In this regard, a pre-sample of 10 runs is first performed to calculate the sample size required for validation. Average waiting times must be collected in each run for verifying whether the simulated model is statistically equivalent to the real-world system. A comparison test between means/medians can be employed for this particular aim. If the resulting p-value is lower than the alpha level (α = 0.05), the simulated model is considered inappropriate for representing the real emergency care system; otherwise, it can be used for performance analysis and ECN design.

iii) *ECN configuration*: The next step is to create an ECN that satisfies the waiting time standards and conditions defined in the previous phases. The performance of the proposed ECN is statistically compared with the current emergency care system. If the p-value is higher than the alpha level (α = 0.05), the ECN is concluded to be satisfactory for reducing the waiting time; otherwise, it should be revised, improved, and reassessed before operation in the real scenario.

### Phase 3: Payment agreement

#### Step 1. Definition of payment policy

The modified collateral payment model $\forall S\left(Nv\left(s\right)\right)=\left[\frac{M\left[1+r\right]}{1+\gamma \theta }\right]$ proposed by Barrios, Caballero, and Sánchez [14] is adopted in this approach. Here, the payment assignment is subject to the characteristic function *N* = {*EN*_1_, *EN*_2_,…, *EN*_m_} where *EN*_*i*_ represents the i-esim emergency node integrating a set of *m* nodes. The nodes are classified into: hospitals and POCs. The payment function covers a collaborative game (2,v):P→R where *M* denotes the amount of payment per admission that is provided to the coalition *S* depending on the health insurance company that the patient is affiliated to. On the other hand, γ and θ are constants that symbolize the contribution of each admission type to the total emergency visits. “γ” represents the percentage of 4-level-triage patients while “θ” denotes the percentage of 5-level-triage patients. The present approach only focuses on these categories due to the following reasons: i) The majority of ED patients are graded as low risk (triage levels 4–5) [52] and ii) These patients can be immediately transferred to another node since their risk of developing more severe complications (including death) is null or very low. Ultimately, “*r*” indicates the correlation between the waiting time of the node receiving the transferred ED patients (*WT*_*i*_) and the number of admitted ED patients (*nap*_*i*_). This measure is adopted to compensate those nodes whose waiting time is affected during the collaboration.

### A case study of a public ECN

This chapter presents an application of the proposed methodology in a South American emergency care system integrated by 2 hospitals and 8 POCs. A detailed description of the case study is provided in each step to fully encompass the different key aspects that should be taken into account by practitioners and healthcare managers when designing in-time and economically sustainable ECNs.

### ECN characterization and performance diagnosis in terms of waiting time

The first step is to properly characterize the EDs that can integrate the ECN and analyze the waiting time that patients may experience when admitted in the above-mentioned emergency care system. In particular, two types of nodes were identified: Hospitals and POCs. On one hand, POCs are nodes that lie at a distance of 1,500 meters from the urban zones and operate 24 hours per day. On the other hand, hospitals are the nodes with the highest installed capacity. They are, however, located further away from the community if compared to POCs. A matrix containing the transfer times (The times between nodes considering normal traffic conditions with no unforeseen eventualities) inherent to each particular slot. An example is provided in Table 1.

**Table 1:** Transfer times between nodes for afternoon slot (in minutes).

	H1	H2	POC1	POC2	POC3	POC4	POC5	POC6	POC7	POC8
H1	NA	11	6	22	10	4	8	14	13	14
H2	12	NA	18	29	19	12	13	13	9	25
POC1	6	19	NA	19	7	10	11	16	19	12
POC2	16	27	11	NA	10	20	20	20	25	22
POC3	9	19	8	14	NA	13	16	23	22	18
POC4	4	11	9	25	11	NA	10	13	12	18
POC5	8	13	11	28	15	9	NA	12	13	17
POC6	14	14	18	35	24	15	11	NA	8	17
POC7	14	9	19	32	20	12	15	6	NA	22
POC8	14	24	12	13	18	18	13	16	21	NA

Table 2 characterizes hospitals and POCs in terms of complexity level, installed capacity (beds), associated health insurance companies, demand, and waiting time. In particular, it can be observed that POC4 does not provide emergency care although it will be enabled in the future for improving the ECN timeliness. It can be also concluded that H2 is the node with the highest average and variable demand per semester (μ = 65,908.5 patients; σ^2^ = 41,137). Additionally, H2 has the lowest waiting time compared to the rest of the nodes (μ = 3.71 minutes; σ^2^ = 0.31) which evidences that its emergency care configuration effectively responds to the current demand. On a different tack, patients who ask for emergency care in H1 and POCs are expected to wait for more than the standard (30 minutes). Hence, cluster managers should focus on improving the timeliness of such nodes to minimize the operational drawbacks that may occur during the ECN operation.

**Table 2:** Characterization of nodes potentially integrating the ECN.

Node	Complexity Level	Installed capacity (beds)	Insurance companies	Demand (patients/semester)		Waiting time (min/patient)	
				μ	σ^2^	μ	σ^2^
**H1**	1	12	S, BU, MS, COM, COO, SV	10,255.72	36.71	182.96	10,610.38
**H2**	2–3	35	S, BU, MS, COM, COO, SV	65,908.5	41,137	3.71	0.31
**POC1**	2	11	S, BU, MS, COM, COO, SV	11,521.08	55.26	188.36	9,854.44
**POC2**	2	13	S, BU, MS, COM, COO, SV	8,775.5	23.83	177.32	10,530.05
**POC3**	2	11	S, BU, MS, COM, COO, SV	8,370.25	20.94	184.50	11,427.58
**POC4**	2–3	NA	NA	NA	NA	NA	NA
**POC5**	2	14	S, BU, MS, COM, COO, SV	14,060.76	49.08	173.68	11,170.08
**POC6**	2	11	S, BU, MS, COM, COO, SV	8,339.89	42.73	190.02	10,269.51
**POC7**	2	12	S, BU, MS, COM, COO, SV	10,260.61	47.71	182.07	9,795.49
**POC8**	2	11	S, BU, MS, COM, COO, SV	8,355.67	41.67	187.15	10,519.84

### Application of LSS for improving the timeliness of each emergency care node

The LSS is applied before the collaboration to reduce the waiting time of ED nodes and thereby, minimizing potential failures and operational drawbacks that may occur during emergency care and patient transfer flows. The LSS project implemented in POC3 has been taken as an example to describe how the timeliness can be improved through the DMAIC cycle:

#### Define

Initially, a six-sigma team composed of six members (Quality manager, 2 Quality assistants, financial manager, financial assistant, and General Manager) was established to support the LSS implementation. The group was guided by two industrial engineers with a black belt level and wide experience in the execution of LSS projects.

**Fig 2:**
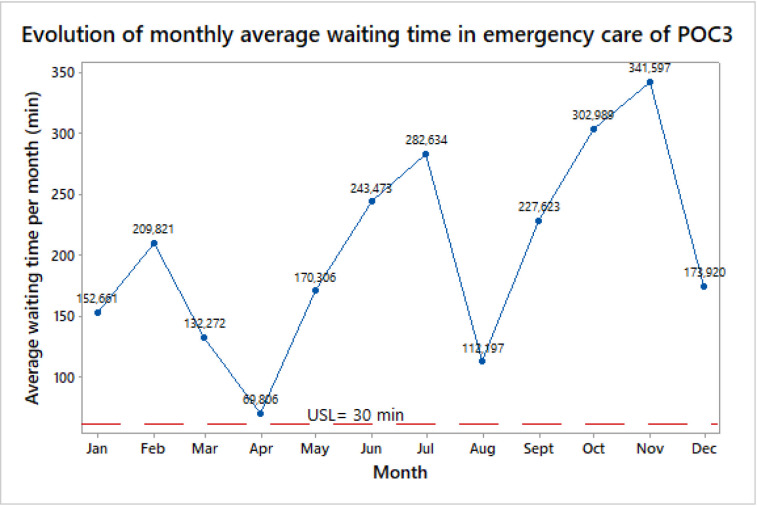
Average waiting time in emergency care–POC3.

A line chart was used to verify the current performance of POC3 in terms of waiting time. Minitab 17 ® software was employed for this particular aim. In this case, the average waiting time was found to be 201.6 min with a standard deviation of 81.6 min. In addition, Fig 2 indicates that the standard Upper Specification Limit (USL) has not been satisfied in the last operational year. POC3 then needs serious interventions to diminish the waiting time and consequently minimize patient dissatisfaction, overcrowding, operational costs, and the development of more severe complications related to patients’ health.

**Fig 3:**
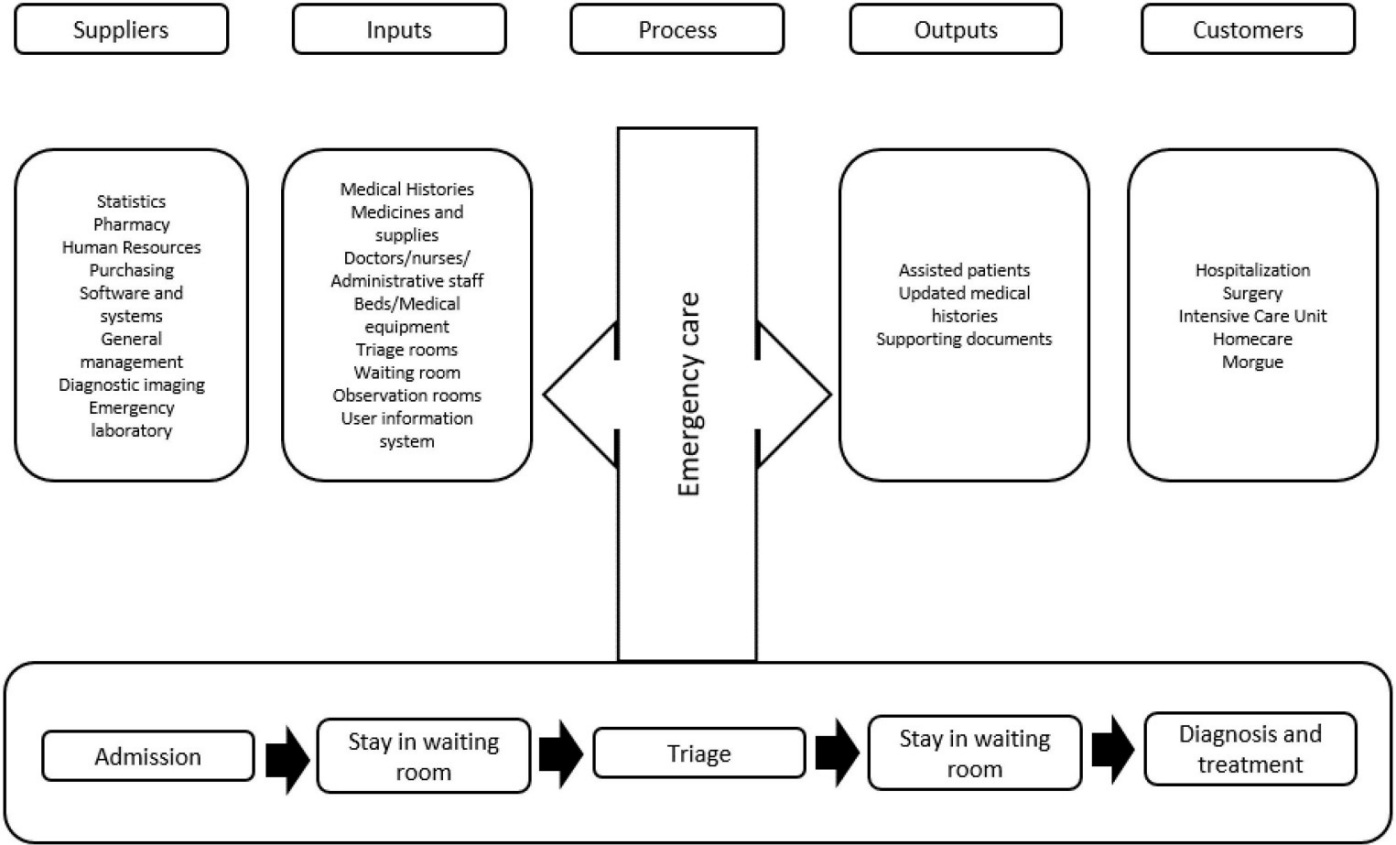
SIPOC diagram for emergency care in POC3.

Considering the information above, a project charter was established. In this application, various benefits for the stakeholders (emergency patients, government, the board of directors, and clinical staff) and two key performance indexes (average waiting time in ER; operational cost per admission) were defined. In addition, the objectives were discussed to obtain formal approval from the sponsor and ethics committee before implementation. Afterward, a SIPOC diagram was created to identify the main activities of emergency care and the interactions with other departments within the node (Fig 3). By using this graph, different pathways and two instances of patients waiting for their physician (Potential intervention point) are observed; in addition, multiple and complex interactions take place in this node which is consistent with Kaushal *et al*. [53] and Kuo *et al*. [54].

#### Measure

The times of registration and initial contact with ER physician corresponding to the last operational year of POC3 (n = 16,741 admissions) were gathered using the Data Warehouse administered by the Ministry of Health and Social Protection. After this, waiting times were estimated with the support of Minitab 17® software. Then, a Ryan-Joiner test was performed to verify the normality of these data. With a *p-value > 0*.*10*, there is then sufficient evidence to conclude that waiting times follow a normal distribution.

**Fig 4:**
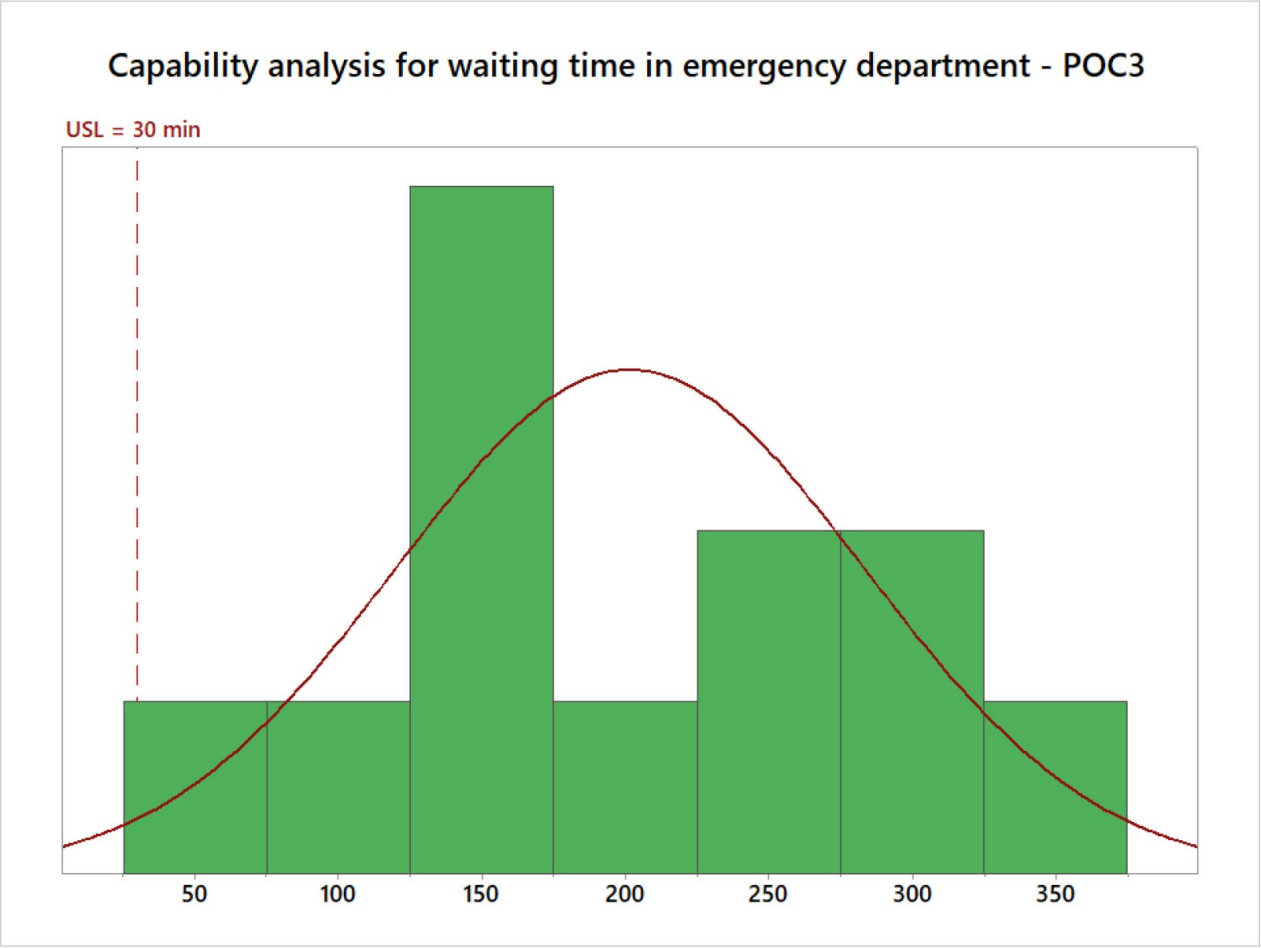
Capability analysis for waiting time in the emergency department–POC3.

Afterwards, a capability study was undertaken to establish how capable POC3 is to meet the standard (Fig 4). Table 3 depicts the six sigma indicators that helped decision-makers to understand the current status of POC3 in terms of waiting time. First, *Cps* was found to be -0.73 which indicates that POC3 is not capable to comply with the standard. As the process is categorized in the lowest performance range, serious and profound changes are therefore necessary for improvement. This is consistent with the short-term sigma level (-2.10) which also reveals that the process is catastrophic and requires immediate intervention. In other words, it is estimated that 985,306.3 in every 1,000,000 patients will experience waiting times over 30 min.

**Table 3:** Six sigma indicators for waiting time in the emergency care–POC3.

*Waiting time in the emergency department–POC3*			
*USL (min)*	30	*Efficiency*	1.47%
*Mean*	201.6	*Cps*	-0.73
*Standard deviation*	81.6	*PPM > USL*	985,306.3
*Zu*	-2.10	*Short-term sigma level*	-2.10
*P(error)*	98.53%	*Long-term sigma level*	-3.60

#### Analyze

**Fig 5:**
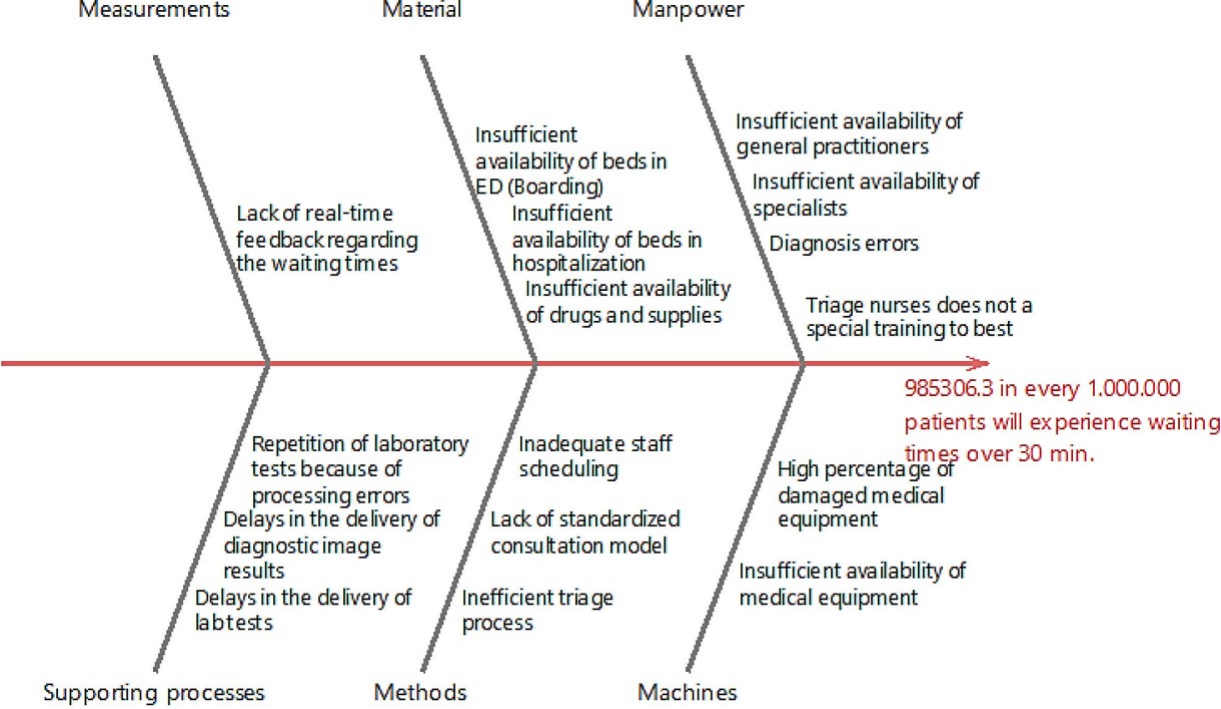
Fishbone diagram for establishing the potential causes of extended waiting times in the emergency department of POC3.

Considering the above-mentioned results, the emergency department in POC3 requires the development of improvement plans aiming at reducing the current waiting time experienced by patients before the first contact with the physician. In this regard, a fishbone diagram was created to find the root causes of the problem (Fig 5).

The diagram evidences all the causes that may generate extended waiting times in the emergency department of POC3. The potential causes were identified with the aid of the Six-sigma team so that focused and further investigation can be made on the process. Statistical significance tests (α = 0.05) revealed that the *average waiting time for the delivery of diagnostic imaging to ED* was found to meaningfully contribute to the *increased waiting time experienced within the ED–POC3* (*p-value* = 0.000; *β =* 1.167; CL = 0.95). The potential influence of the average laboratory turnaround time was also explored. Similar to diagnostic imaging, a significant association was detected (*p-value* = 0.004; *β =* 0.734; CL = 0.95) [55]. On the other hand, it was concluded that the *percentage of damaged equipment* also leads to the problem (*p-value* = 0.000; *β =* 937.8; CL = 0.95). These findings suggest that the untimely provision of diagnostic aids increases the length of stay and bed occupancy within the emergency department which, in the meantime, increases the waiting time experienced by the recently admitted patients [56–57]. It is also evidenced that the effective provision of emergency care highly depends on the suitable management of interactions between the ED and other departments. In this regard, POC3 managers should focus on improving the response time of supporting departments so that diagnosis and treatment processes can be expedited within the ED. On a different tack, *insufficient availability of beds in hospitalization*, *beds in ED*, *drugs and supplies*, *general practitioners*, *specialists*, *and medical equipment* were also found as significant regarding the extended waiting time (*p-value <* 0.005).

#### Improve

Considering the analysis outputs, the six-sigma team proceeded with the creation of improvement strategies aiming at lowering the current waiting time experienced by patients. In this respect, four actions were proposed and instituted: i) Reconfiguration of work shifts according to the workload needs and the available number of laboratorians; ii) Transferring of specimens to the lab in batches so that the first batch can be processed whilst the second batch is collected; iii) A scheduling program that assigns radiologists to read studies according to a priority level that considers both patient triage category and delay; and iv) Removal of non-value activities during the reading of imaging studies through the use of Value Stream Mapping and other lean manufacturing techniques.

**Fig 6:**
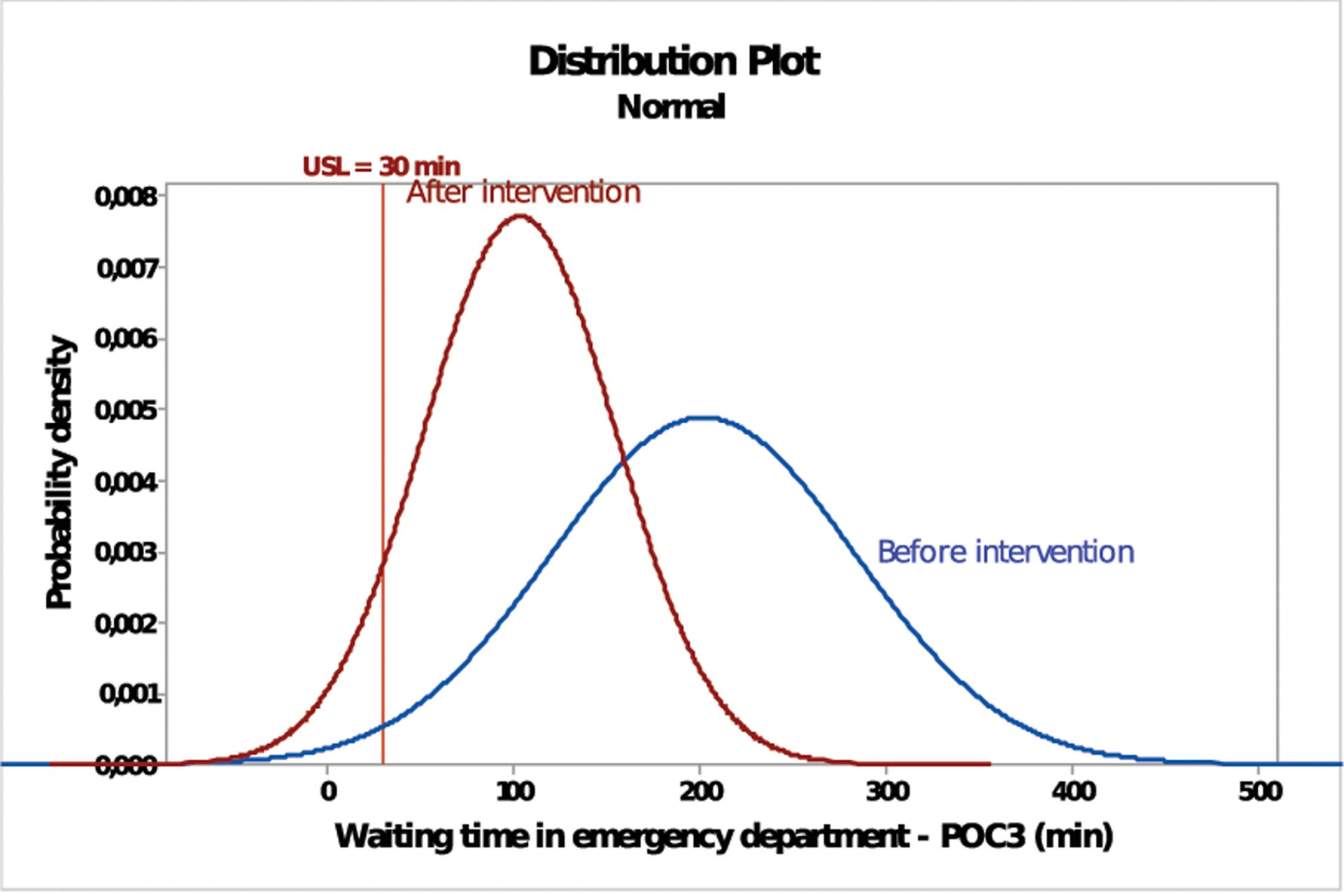
Before-after intervention in POC3.

After a 3-month intervention, the collected waiting times were processed with the aid of Minitab 17® software to evaluate whether the implemented changes were satisfactory. The results are summarized in Fig 6. In detail, the Cps (-0.45) has increased compared to the initial status; nonetheless, the process is not yet capable of meeting the government standards. This means that the implemented changes are not enough for propelling the ED to the desired performance. This is confirmed through the short-term sigma level (-1.41) and PPM (921,329) which evidenced a slight improve but also a catastrophic emergency care in terms of waiting time. Consequently, the efficiency passed from 1.47% to 7.87% whilst the long-term sigma level increased to -2.91.

Similar to POC3, LSS was applied in each of the nodes (except H2 which has a short-term sigma level higher than 6) potentially integrating the ECN. The results in terms of average and standard deviation of waiting time, short-term sigma level, and PPM have been enlisted in Table 4. To sum up, all the hospitals and POCs improved their waiting time for emergency care. However, some nodes (POC1, POC2, POC3, POC4, and H1) still evidence a catastrophic process (PPM > 800,000; short-term sigma level < 0). Therefore, some changes are still necessary to diminish the patients’ stay in waiting rooms. In this respect, improvement strategies regarding installed capacity and availability of resources may be explored through collaborative scenarios as detailed in the following steps of this implementation.

**Table 4:** Summary of results achieved through LSS projects in potential ECN nodes (except H2).

Node		H1	POC1	POC2	POC3	POC4	POC5	POC6	POC7	POC8
**Short-term σ level**		-0.69	-1.66	-1.23	-1.41	-1.42	0.31	2.51	3.49	3.43
**PPM**		755,915	951,187	890,103	921,329	922,368	376,994	5,979	237	302
**Waiting time**	**Μ**	69.9	126.03	89.87	103.1	126.98	26.11	17.14	13.89	13.20
	**σ**^**2**^	3,305.3	3,361	2,381.3	2,671.1	4,656.9	153.74	26.18	21.25	23.99

#### Control

After implementing the improvement strategies and verifying their effectiveness in the wild, the Quality department proceeded with incorporating these changes into the Quality Management System. Besides, X-R control charts for individual observations were designed to monitor the average and variation of waiting times experienced by the emergency patients. All these activities were undertaken to keep the performance achieved during the LSS project and consequently avoid a potential decline in the timeliness of emergency care when collaborating with hospitals and POCs.

### Establishment of the ECN legal framework

It was evident that further lowering of waiting times is still necessary. In this sense, a collaboration scheme may be a good option considering the restricted budget that prevents nodes from expanding their installed capacity. In this respect, an important step is the identification of the regulations governing the provision of emergency care in the region where the ECN will take place. Such regulations become a critical to satisfaction that must be taken into account by decision makers when designing the ECN. The related laws, regulations, and resolutions have been enlisted and shortly described in S1 Table. The next step will be then to determine how the ECN can incorporate these insights in its daily operations so that legal requirements can be fully fulfilled.

**Fig 7:**
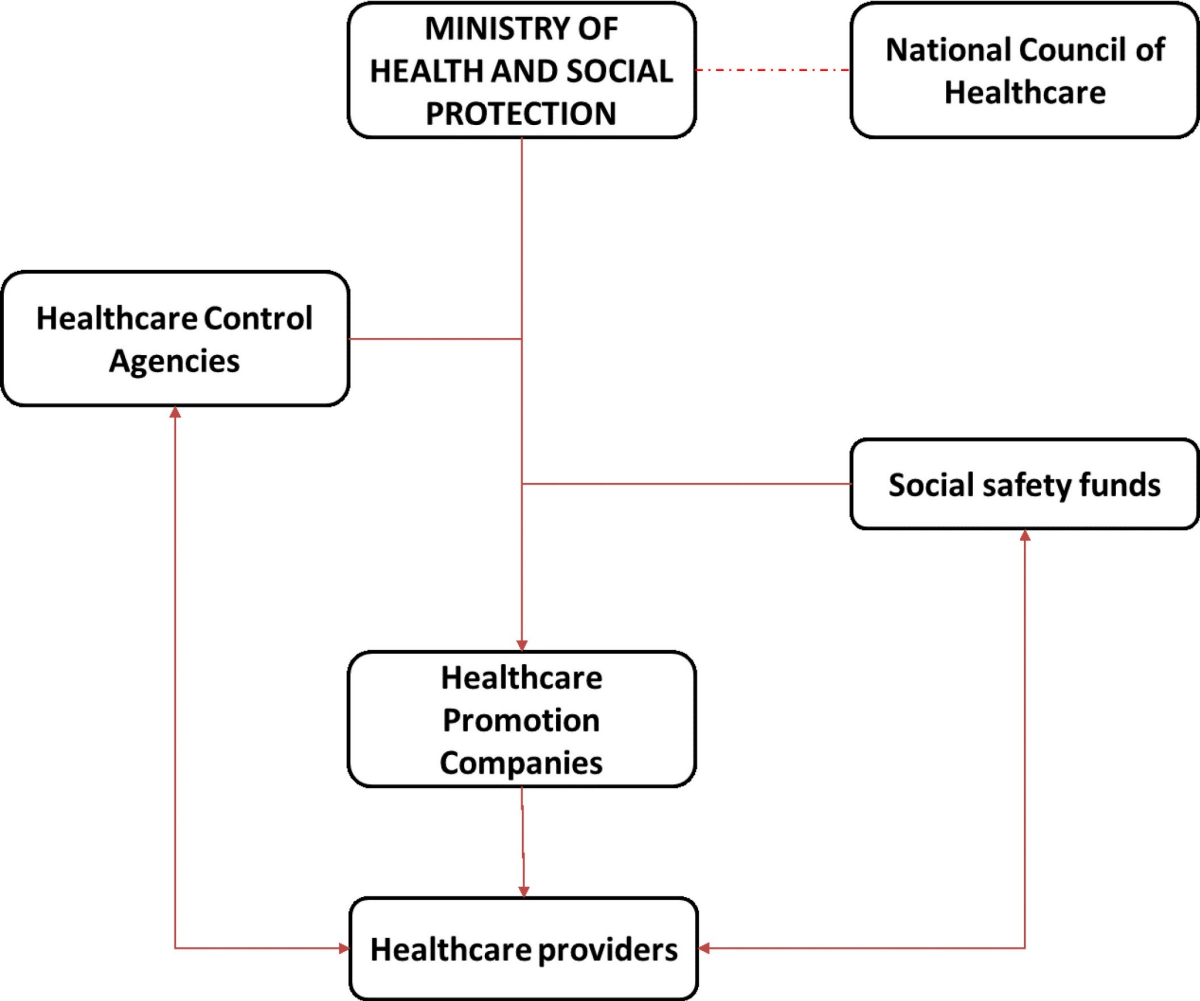
Configuration of healthcare system.

Another important aspect to be considered within the legal framework is the current configuration of the healthcare system (Fig 7). This highly affects the flows of collaboration and information within the ECN and must be thus considered during the design process. Considering above, the ECN will be then involved in a very complex administrative and legal structure whose demands and regulations must be properly addressed to ensure a correct and efficient flow of operations, information, and earnings.

### Definition of network’s target population

Table 5 presents the number of affiliated patients to be potentially admitted within the ECN (1,229,996). In detail, MS is the insurance company with the highest portion of patients (n = 371,274–30.35%) while SV has the smallest participation (n = 92,887–7.59%). On the other hand, it is also necessary to consider the patients who are under a special regime (n = 37,314) or those who are not covered by social security (n = 47,973). This lies in the fact that, in accordance with the international regulations, “patient dumping” is not anymore permitted. In total, it is estimated that 1,315,283 patients will have access to the emergency services provided by the ECN.

**Table 5:** Number of affiliated patients to healthcare promotion companies.

Health Insurance companies	S	BU	MS	COM	COO	SV	TOTAL
**Number of affiliated patients**	240,68	159,033	371,274	106,386	252,736	92,887	**1,229,996**

### Design of the ECN strategic platform

Taking into account a target population of 1,315,283 patients, the ECN legal framework and the current performance of hospitals and POCs, we proceeded with the definition of the ECN strategic platform. Initially, the stakeholders and their critical-to-satisfaction (CTS) factors were identified (S2 Table) through the VOC analysis. In summary, 17 stakeholders were found to be associated with the ECN functioning. Besides, the most popular expectations (CTS factors) were: *correct and complete provision of patients’ information* (n = 15; 88.23%), *nimble attention* (n = 7; 41.17%), and *respect and support from physicians and nurses* (n = 6; 35.29%). Such needs must be then highly prioritized by the managers so that stakeholders can be fully satisfied during ECN operation. This is complementary to the aforementioned legal framework and payment model that must be also incorporated into the ECN design. Considering these findings, the mission was defined as: *Our mission is to deliver nimble and high-quality emergency care for our patients through an efficient*, *integrated*, *and financially sustainable network of hospitals and points of care*. Besides, the vision was established as: *In 2*,*020*, *we will be recognized as the first regionalized*, *coordinated*, *and accountable emergency care network throughout the country*. Also, the board of directors defined four strategic goals supporting the accomplishment of mission and vision: *Aim 1*: To monitor the timeliness of care and ensure that patients do not experience excessive waiting times in ECN nodes; *Aim 2*: To ensure equitable distribution of payments within the ECN; *Aim 3*: To implement research projects targeting optimal resource allocation, patient flow, and information transfer; and *Aim 4*: To ensure the effective link among ECN nodes through a central information platform that facilitate decision-making and planning.

Lately, the ECN corporative values were defined considering the stakeholders’ expectations, external conditions, mission, and vision: *Collaboration*, *professionalism*, *evidence-based decision making*, *innovation*, *service excellence*, and *integrity*. Such values must be considered during the development of governance arrangements, service protocols, and policies, aspects that will be further analyzed in the next chapter.

### Development of governance arrangements, service protocols, and policies

**Fig 8:**
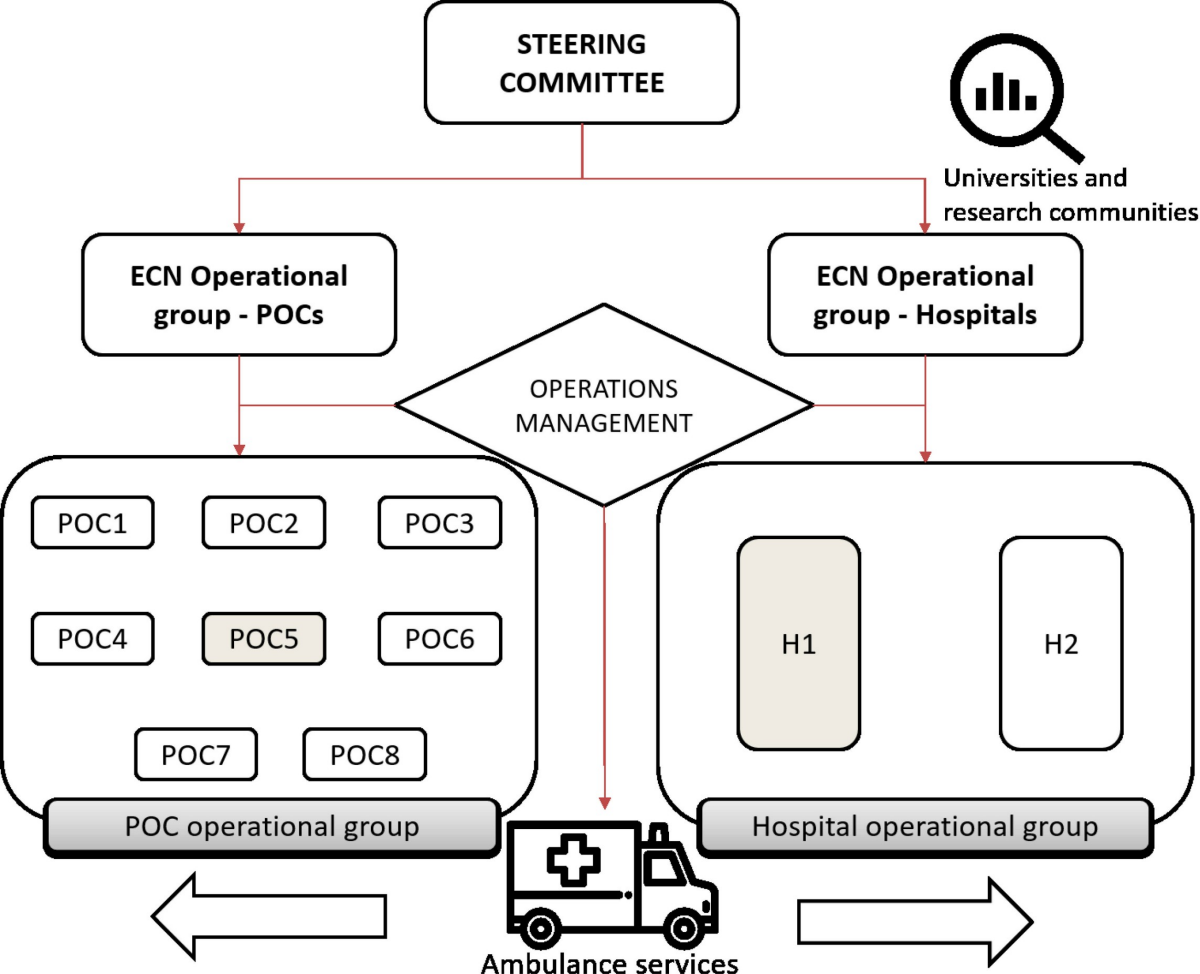
ECN governance structure.

Fig 8 illustrates the governance structure to be adopted for regulating the ECN operations. The activities regulating this structure include: *clinical audit*, *guideline implementation*, *measurement of KPIs*, and *risk management*. These tasks should be overseen by the *Steering Committee* which is also called to: i) drive improvements related to the quality, and cost-effectiveness of patient care, ii) steward resources within ECN and each node, iii) establish responsibility, authority, and accountability across the ECN, iv) propel the coherent integration among ECN nodes, v) review external conditions and national guidelines that may affect the ECN functioning, vi) supervise workforce planning across the ECN, vii) build relationships with other healthcare bodies, and viii) ensure preparedness across the ECN.

On a different tack, the *ECN operational groups* (POCs and hospitals) are led by 1 network coordinator each. Such groups are comprised of: ED managers, full-time consultants, and representatives from patients’ association, ambulance services, operations management department, associated healthcare services (laboratories, hospitalization, intensive care unit, supplies and drug management, and diagnostic imaging department), ECN physicians, and ECN nurses. The functions of these groups are the following: i) supervise the implementation of improvement strategies designed by the *Steering Committee*, ii) undertake audits and examine data reports, iii) progress emergency care development and staffing matters at ECN level, iv) oversee risk management, clinical education, clinical audit, and other governance activities, v) propel effective communication among ECN nodes, vi) advise the *Steering Committee* regarding findings and aspects of relevance, and vii) govern interface flows.

On the other hand, each ECN node must continuously: i) verify advance in achieving KPI targets, ii) address staffing matters, iii) guarantee the stakeholders’ participation in decision-making process, iv) monitor adverse events and ECN risks, v) revise feedback from the respective *ECN operational group*, vi) implement national guidelines related to emergency care, vii) implement educational programs regarding the ECN functioning and the correct use of emergency services.

After defining the ECN governance structure, roles, authority, and responsibilities; the protocols and policies related to the provision of emergency care were established for implementation within the network (Table 6). To sum up, 6 types of protocols were identified, categorized (indoors/outdoors), and related to the pertinent domains of emergency care. Most of them (5), were classified as “indoors” while only 2 were considered as “outdoors”. When relating these protocols to the ED domains, *procedures and protocols*, *human resources*, and *quality* were found to be influencing in the development of all the service regulations.

**Table 6:** Service protocols within ECN.

Protocol	Category		Related domains
	Indoors	Outdoors	
Guide for emergency management	X	X	PP, ME, I, SMA, HR, SP, and Q.
Guide for good practices in patient safety	X		PS, I, HR, PP, SMA, ME, SP, and Q.
Basic guide for pre-hospital care		X	PS, HR, PP, SMA, ME, I, and Q.
Biosecurity, pegirase, cleaning and disinfection, and sex abuse	X		PS, HR, PP, SMA, ME, I, PS, and Q.
Guide for healthcare monitoring	X		PP, HR, SP, PS, and Q.
Guide for patients’ referral and back-referral	X	X	PP, ME, SMA, HR, PS, and Q.

### Definition of ECN risks

S3 Table enlists the failures that may occur during ECN operation (specifically related to waiting time) along with their severity (S), frequency (F), detection (D), and risk priority number (RPN) [58]. The risks with an RPN > 125 and significant severity (8–10) have been denoted with double asterisk (**) while risks with high RPN (> 125) and no meaningful impact (S < 7) were marked with one asterisk. Both types of risk must be prioritized for immediate intervention through corrective and preventive plans as detailed in S4 Table. Based on the information provided by FMEA, the most critical failures (wrong triage classification and delay to triage; RPN = 450**) are related to *higher mortality rate*, no controls and frequent potential causes (misjudgment of the physical symptoms and delay during triage classification). This evidences that the triage processes are the major highest-risk sources within the ECN. Being aware of this situation, it is necessary to train doctors to categorize patients correctly, implement p control charts to monitor the percentage of correctly classified patients, and apply the Value Stream Mapping (VSM) to detect and eliminate non-value activities during the triage process.

### Development of a DES model to establish the ECN configuration

The next step is to design a virtual model representing how the ECN will operate within the aforedescribed context. Such a model will serve as a platform for i) deciding whether a patient should be transferred to another node, ii) identifying which node can provide the most timely emergency care considering transfer times, iii) assessing the balance between the current installed capacity and demand, iv) evaluating new scheduling policies, and v) determining ambulance service requirements based on transferring needs. The model robustness, however, lays on the correct deployment of the step-by-step procedure explained in Phase 2. The results of applying such a procedure in our case study network are further detailed in the following paragraphs.

#### Input data analysis

Data corresponding to 8 variables were collected for representing the real performance of each node (Table 7). Run tests (α = 0.05) were initially performed to determine if these variables were random in each node. The results obtained from H1 are provided as an example (Table 7). In this case, the p-values and *k* metrics provided enough support for accepting the independence hypothesis. This pattern was also found to be valid in the rest of ECN nodes.

**Table 7:** Results of randomness tests in H1.

Process variable	K	P-value
Time between arrivals (min)	33.349	0.387
Triage time per patient (min)	3.495	0.235
Admission time (min)	7.482	0.553
Bed preparation time (min)	7.509	0.691
Nursing assistance time (min)	6.499	0.223
Physician assessment time (min)	16.025	0.162
Treatment time (min)	251.886	0.681

After verifying the randomness nature of these variables, Kruskal-Wallis tests (α = 0.05) were undertaken to identify potential sub-groups of data. In H1 (Table 8), mostly variables were found to be homogeneous except “time between arrivals” (p-value = 0). This outcome is explained by the presence of different demand patterns throughout time. Specifically, the weekday and period of arrival were found to explain the variation observed in the number of emergency admissions (p-value < 0.001). This means that a statistical expression must be defined for representing the time between arrivals corresponding to each combination “weekday-time slot”; in the meantime, one probability distribution is sufficient for describing the homogeneous variables considered in this network. The above-mentioned conclusions were also derived from the other hospitals and POCs.

**Table 8:** Results of homogeneity tests in H1.

Process variable	P-value	Conclusion
Time between arrivals (min)	0.000	Heterogeneous
Triage time per patient (min)	>0.15	Homogeneous
Admission time (min)	>0.10	Homogeneous
Bed preparation time (min)	>0.15	Homogeneous
Nursing assistance time (min)	>0.15	Homogeneous
Physician assessment time (min)	>0.15	Homogeneous
Treatment time (min)	0.363	Homogeneous

Chi-squared tests (α = 0.05) were then implemented to find the statistical distribution that better fits each variable. H1 was again selected to evidence the application of these tests (S5 Table). Following this, an ANOVA F-test (α = 0.05) was performed to determine whether the “time between arrivals” needed to be divided in time slots. As a result, 21 pipelines conditioned by the combination of seven weekdays (M: Monday, Tu: Tuesday; W: Wednesday; Th: Thursday; F: Friday; Sa: Saturday; Su: Sunday) and three time slots: P1 (12:00 am– 8:00 am), P2 (8:00 am– 4:00 pm), and P3 (4:00 pm– 12:00 am) were identified (p-value < 0.005); thereby confirming the heterogeneous nature of “time between arrivals” throughout the weekdays and day shifts. This process was repeated until defining the probability distributions of all variables affecting the ECN operation.

#### Creation and validation of a DES model

**Fig 9:**
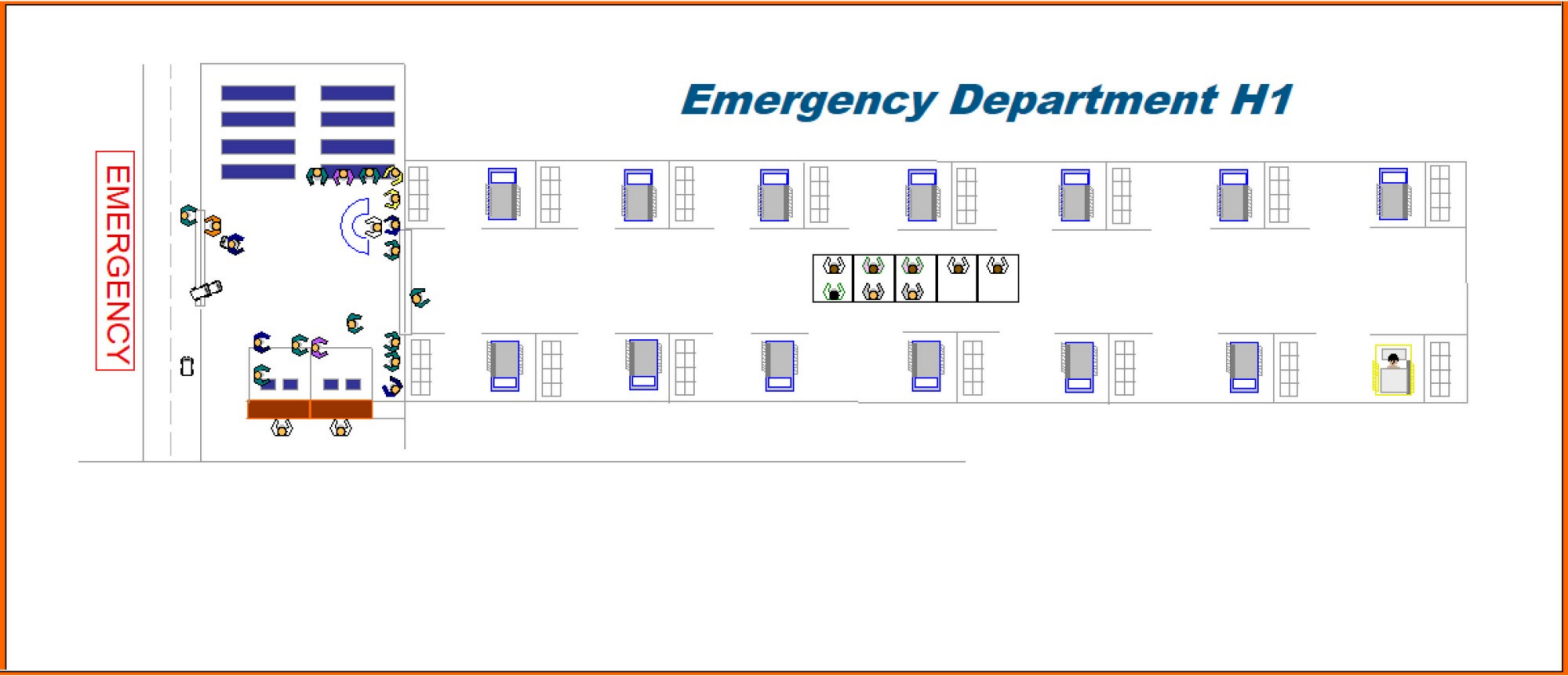
Simulation model of emergency department H1.

A DES model was created through Arena® 15 to provide a virtual representation of the current emergency care system and internal configuration of hospitals and POCs (Fig 9). The model incorporated the results of previous steps including the input analysis and system characterization. Given the continuous operation of emergency departments, a replication length time (365 days– 24 hours per day) was assumed during the simulation. Also, the warm-up period was defined to be 100 days since, at this point, the variation of the blocking probability was found to be near 0 (95% CI [1.85%; 1.89%]); thereby denoting that the steady state of the system has been achieved. Ten replications were later carried out for estimating the number of iterations that should be finally run for validating the simulated system. In this case, 4,532 replications were found to be necessary for representing the waits experienced by patients within this network. After gathering the waiting times derived from each replication, we proceeded to evaluate the equivalence hypothesis $\left(H_{0}:\mu =58.9\;\frac{min}{admission}||H_{0}:\mu \neq 58.9\frac{min}{admission}\right)$. In this case, the 1-sample t test (Confidence level = 0.95) evidenced that the simulated model is statistically comparable with the real system (*p*-value = 0.586; T = 0.54; 95%CI [62.37–65.87] min). This outcome was corroborated through a one-sample variance test whose *p*-value (0.099) confirms that the model is suitable to support performance analysis and ECN design.

#### ECN configuration

It is noteworthy that hospitals and POCs involved in the current emergency care system do not consider transferring patients admitted in their emergency rooms. In other words, each node takes care of their patients no matter how much time they have to wait before diagnosis and treatment. Our proposal is then to design an ECN where hospitals and POCs can collaborate so that patient waiting times can be plummeted while ensuring financial sustainability. To do these, several policies should be adopted into the network: i) Only 4-level-triage and 5-level-triage patients can be transferred from one node to the other; ii) A patient is transferred if the waiting time offered by the origin node is higher than the sum between the transfer time and the waiting time expected in destination node; otherwise, the patient should be treated in the origin node. If there are several transferring alternatives, managers should select the alternative with the lowest sum; iii) Conditions regarding emergency care provision and triage classification system must be fulfilled by nodes to interact within the network; iv) Both origin and destination nodes must hold an agreement with the healthcare promotion company to which the patient belongs. If this condition is not met, the patient cannot be transferred; v) “Patient dumping” is not permitted in this network; vi) Correct and complete provision of patients’ information, nimble attention, and respect/support from physicians and nurses must be granted during ECN operation; vii) Every participating node must adopt a DSS to verify if another node can provide faster emergency care considering transfer times. The DSS is also called to support the transferring process if this is finally approved by the Operations Management department; viii) Participating nodes are required to assume the ECN governance structure during operation; and ix) Hospitals and POCs must adhere to the recommendations derived from FMEA application to effectively deal with the predefined risks.

The ECN incorporating all these policies was later modeled, simulated, and assessed to define whether it was effective for minimizing waiting times. Table 9 presents the door-to-physician times that may be experienced by patients if hospitals and POCs operate collaboratively as a network. From this table, it can be inferred that the waiting time mean and variance were minimized in H1, POC1, POC2, POC3, and POC4 nodes; while these metrics increased in H2, POC5, POC6, POC7, and POC8. The next step was to perform a before-and-after analysis for verifying the effectiveness of the network if implemented in the wild.

**Table 9:** Projected waiting times (if the ECN is implemented).

Node		H1	H2	POC1	POC2	POC3	POC4	POC5	POC6	POC7	POC8
**Waiting time**	**Μ**	48.23	4.19	80.65	70.99	76.29	96.50	29.5	19.53	15.69	15.04
	**σ**^**2**^	2,082.33	0.35	2,083.82	1,857.41	1,762.92	2,840.7	173.72	29.84	24.01	27.34

The null and alternative hypothesis associated to this analysis are as follows: $Ho:\;n_{ECN-}n_{c}=0||Ha:\;n_{ECN-}n_{c}<0$. Here, *n*_*ECN*_ denotes the median waiting time experienced by emergency patients if the ECN is implemented while *n*_*C*_ represents the median ED waiting time experienced by patients under the current configuration. Given the non-normality of *n*_*ECN*_ and *n*_*C*_, a non-parametric comparison test (in this case, Mann Whitney) was decided to be applied (using Minitab 19® software) for validating the hypothesis. In this case, the Mann-Whitney test provided sufficient support to conclude that the ECN is satisfactory for lowering the ED waiting time (*p*-value = 0; W = 17,791,765.5; 95%D[-9.08; -6.71]). In particular, if the ECN is implemented, the patients may experience a faster emergency care with an expected reduction of waiting times ranging from 6.71 min and 9.08 min. On the other hand, a paired t-test (using Minitab 19® software) was undertaken to verify whether the median ED bed occupancy would increase after implementing the proposed framework ($Ho:\;n_{BO\left(ECN\right)-}n_{BO\left(c\right)}=0||Ha:\;n_{BO\left(ECN\right)-}n_{BO\left(c\right)}<0$). Here, *n*_*BO*(*ECN*)_ symbolizes the median bed occupancy in POCs and hospitals if the ECN is implemented while *n*_*BO*(*C*)_ denotes the median bed occupancy in POCs and hospitals under the current configuration. The results revealed that hospitals and POCs would have resource utilization rates (*p*-value = 0; T = 5.85; 95%D [8.06%; 18.21%]) ranging from 8.06% and 18.21% increase (Confidence level = 95%) if the proposed network design is adopted. In light of these results, the proposed methodology is hence considered as effective for ensuring not only the timeliness of the ECN here designed but the resource usage within each node.

### Definition of payment policy

After verifying the advantages of collaboration in terms of waiting times, it is necessary to ensure the efficient and equitable distribution of payments among participant hospitals and POCs either origin or destination nodes. The collateral payment model is proposed within this study to deal with this challenge. One of the variables influencing the model is *M* which denotes the amount of payment that is provided to the coalition *S* when a patient is transferred to a destination node. The unit utility value depends on the healthcare promotion company that the patient is affiliated to (Table 10). Other variables of interest in this scheme are γ (percentage of 4-level-triage patients) and θ (percentage of 5-level-triage patients). In this network, γ and θ were found to be 0.19 and 0.46 respectively. After defining these parameters, we proceeded to establish the payment distribution between the origin and destination nodes (Table 11).

**Table 10:** Unit utility values agreed with healthcare promotion companies.

Healthcare promotion company	S	BU	MS	COM	COO	SV
**M (Unit utility value) in US$**	10.34	4.91	4.91	4.91	5.11	9.97

**Table 11:** Payment distribution arrangements between origin and destination nodes.

		Destination Node									
		H1	H2	POC1	POC2	POC3	POC4	POC5	POC6	POC7	POC8
**Origin Node**	**H1**	M	A	B	B	B	B	B	B	B	B
	**H2**	C	M	B	B	B	B	B	B	B	B
	**POC1**	C	A	M	B	B	B	B	B	B	B
	**POC2**	C	A	B	M	B	B	B	B	B	B
	**POC3**	C	A	B	B	M	B	B	B	B	B
	**POC4**	C	A	B	B	B	M	B	B	B	B
	**POC5**	C	A	B	B	B	B	M	B	B	B
	**POC6**	C	A	B	B	B	B	B	M	B	B
	**POC7**	C	A	B	B	B	B	B	B	M	B
	**POC8**	C	A	B	B	B	B	B	B	B	M

If the origin and destination nodes are the same, the hospital or POC receives *M*; otherwise, payment arrangements *A*, *B*, or *C* must be applied according to Table 11. The arrangements are described as follows:

- *“A”*—Origin node:$M-Máx\left\{US\$3.92;\frac{M\left(1+r\right)}{1+\gamma \theta }\right\}$ ||Destination node:$Máx\left\{US\$3.92;\frac{M\left(1+r\right)}{1+\gamma \theta }\right\}$
- *“B”*—Origin node: $M-Máx\left\{US\$3.50;\frac{M\left(1+r\right)}{1+\gamma \theta }\right\}$ || Destination node:$Máx\left\{US\$3.50;\frac{M\left(1+r\right)}{1+\gamma \theta }\right\}$
- *“C”*—Origin node:$M-Máx\left\{US\$4.50;\frac{M\left(1+r\right)}{1+\gamma \theta }\right\}$ || Destination node:$Máx\left\{US\$4.50;\frac{M\left(1+r\right)}{1+\gamma \theta }\right\}$

Table 12 specifies how payments have been settled for coalition H1-H2 considering the above-cited collateral model. In this case, transfer flow “p” from H1 to H2 (6,052 patients) was found to be significantly higher compared to the number of remissions taking place from H2 to H1 (450 patients). On the other hand, non-significant differences were detected when comparing the correlation values of H1-H2 and H2-H1 (p-value = 0.123; T = -1.85; 95%D[-0.1145; 0.0185]). Moreover, the low correlation values observed in this coalition (r ≤ 0.152) indicate that transferred ED patients caused slight affectations on waiting times experienced in destination nodes. It is also good to highlight that two different payment arrangements were applied: “A” (H1-H2) and “C” (H2-H1). In the scheme “A”, the destination node (H2) received US$3.92 for patients affiliated to BU, MS, COM, and COO while this rate increased to US$6.36 and US$6.13 when receiving patients from S and SV respectively. A similar pattern was observed upon applying the arrangement “C”. In this case, H1 earned US$7.08 and US$6.25 per S-covered and SV-covered patient correspondingly. Likewise, the lowest payment rate (US$4.5) was adopted when admitting patients from BU, MS, COM, and COO. Such results are mainly due to the combination of low correlation scores and utility values. On a different tack, both H1 and H2 obtained financial gains (H1: US$12,662; H2: US$29,980) from the coalition. This is highly attractive considering the need for ensuring the financial sustainability of nodes while providing timely emergency care to patients.

**Table 12:** Payment distribution for coalition between H1 and H2 (1 year of simulation).

H1-H2							H2-H1						
	S	BU	MS	COM	COO	SV		S	BU	MS	COM	COO	SV
**nap**_**i**_	1,190	788	1,833	529	1,249	463	**nap**_**i**_	89	59	136	39	93	34
**R**	0.015	0.054	0.043	0.046	0.016	0.021	**r**	0.13	0.152	0.024	0.018	0.124	0.035
**P(H1)**	4,735	780	1,814	523	2,485	1,759	P(H1)*	290	24	55	15	56	126
P(H2)*	7,569	3,089	7,185	2,073	4,896	2,856	**P(H2)**	630	265	612	175	418	212
TP(H1) = P(H1) + P(H1)*				US$ 12,662									
TP(H2) = P(H2) + P(H2)*				US$ 29,980									

The payment settlement process was then repeated until obtaining the total profits of each node (Table 13). In this case, H2 and POC8 were found to be the nodes with the highest total gain within the network (US$212,142 and US$77,064 respectively). It is good to highlight that the significant difference (in terms of total profit) observed between H2 and the rest of nodes is explained by the high number of patients transferred to this hospital (31,810) and the increased waiting time resulting from the collaboration (WT_2_ = 4.19; σ^2^ = 0.35). Lately, it is noteworthy that all nodes obtained financial benefits (μ = US$58,152/node) while ensuring the earliest possible emergency care to patients.

**Table 13:** Total profits of nodes after 1-year collaboration.

Node	H1	H2	POC1	POC2	POC3	POC4	POC5	POC6	POC7	POC8
**Total profit (in US$)**	36,067	212,142	24,756	19,132	18,721	8,138	47,73	61,847	75,923	77,064

### Concluding remarks

ECNs are an important alternative to deal with the excessive waiting time perceived by patients requiring emergency care. These structures, however, are complex to design due to the presence of multiple nodes, resources, and collaboration flows. Moreover, they are called to ensure an equitable and efficient distribution of profits within the network considering different utility functions, healthcare promotion companies, and payment arrangements. In this paper, we proposed a three-phase methodology for the effective creation of ECNs. This approach initiated by characterizing and preparing the nodes through lean six-sigma; thereby the network complexity could be meaningfully diminished before collaboration. We then proceeded to design the ECN considering the legal framework, network’s target population, strategic platform, governance arrangements, service protocols, policies, and risks. After this, the ECN configuration was defined using DES. Finally, payments derived from the collaboration were established by applying the collateral model.

From the managerial perspective, our proposed methodology is suitable for providing decision support to policymakers, government authorities, ED administrators, and stakeholders when addressing the following scenarios: i) deciding whether a patient should be transferred to another node, ii) defining the node providing the most timely emergency care considering transfer times, iii) evaluating the balance between the network capacity and demand, iv) assessing staffing policies, v) estimating ambulance service requirements based on transferring needs, and vi) efficiently distributing profits among participant ECN nodes. From the scientific angle, our paper bridged the gap detected in the literature by laying the methodological groundwork required for the creation of new ECNs in a plethora of healthcare contexts [59].

Concerning the scenario under study, an emergency care system integrated by 2 hospitals and 8 POCs, the results revealed that H2 is the node with the highest average and variable demand per semester (μ = 65,908.5 patients; σ^2^ = 41,137) while H2 has the lowest door-to-doctor time compared to the rest of nodes (μ = 3.71 minutes; σ^2^ = 0.31). Overall, patients requiring emergency care in H1 and POCs were found to wait for more than the government target which was corroborated through negative six sigma levels in most cases. Although the efficiency scores were augmented in all nodes using LSS, collaboration practices were concluded to be necessary. Along the path towards the ECN consolidation, it was determined that: i) 1,229,996 patients are projected to be admitted within the ECN, ii) “*correct and complete provision of patients’ information”* (n = 15; 88.23%), “*nimble attention”* (n = 7; 41.17%), and “*respect and support from physicians and nurses”* (n = 6; 35.29%) were found to be the critical to satisfaction, iii) the most critical failures were: wrong triage classification and delay to triage with RPN = 450, and iv) the ECN configuration was found to be satisfactory for lowering the ED waiting time (*p*-value = 0; W = 17,791,765.5; 95%D[-9.08; -6.71]). On a different tack, three payment arrangements were designed as a basis of the collateral payment model. Such a model was concluded to be satisfactory for nodes upon offering good compensation schemes while propelling lower waiting times for patients.

Given the considerable potential of this approach, we plan in the future to incorporate transferring costs and ambulance routing optimization models for increasing the ECN competitiveness. Thereby, more informative and detailed simulations can be provided for assessing more complex scenarios and interactions. It is also intended to contrast our modified collateral payment scheme with other utility distribution models to improve the profit allocation efficiency within the network.

## Supporting information

S1 Table Laws, resolutions, and regulations related to the ECN design. (DOCX)

S2 Table ECN stakeholders and their expectations. (DOCX)

S3 Table Failure mode and effect analysis for ECN operation. (DOCX)

S4 Table Recommended actions for high-RPN failure modes. (DOCX)

S5 Table Results of Goodness-of-fit tests in H1. (DOCX)
