# Supplementary material for: An integrated approach for designing in-time and economically sustainable emergency care networks: A case study in the public sector
Source: PLoS One. 2020 Jun 22;15(6):e0234984. doi: 10.1371/journal.pone.0234984 (PMC7307761; doi:10.1371/journal.pone.0234984)
Supplement: S1 Table — (DOCX) [file pone.0234984.s001.docx]

| Law/Resolution/Agreement | Description | Main insights to consider in ECN design |
| --- | --- | --- |
| Political Constitution | It establishes that healthcare is a public service in charge of the government. In this regard, the government must ensure the access to the promotion, protection, and habilitation of healthcare services. Also, it defines that these services must be provided under the principles of efficiency, solidarity, and universality. Finally, it specifies that healthcare attention must be organized by levels while ensuring the community involvement. | Principles of emergency care:  i) Efficiency  ii) Solidarity  iii) Universality |
| System of Social and Integral Insurance | It indicates that the government is required to establish programs and policies ensuring the access to healthcare services under the principles of efficiency, solidarity, universality, integrality, community involvement, and unit. | Principles of emergency care:  i) Efficiency  ii) Solidarity  iii) Universality  iv) Integrality  v) Unit |
| Mandatory System of Quality Assurance | It points out that healthcare providers must comply with the following conditions: i) Technical-administrative capacity, ii) financial and patrimonial proficiency, and iii) technological-scientific capacity. Besides, it specifies that healthcare services must be provided in an accessible and equitable manner while considering an optimal balance among benefits, risks, and costs. This is to achieve a high satisfaction and loyalty of users. Finally, it involves specific procedures for the Quality information monitoring and management, habilitation, and accreditation. | a) The need for optimal balance among benefits, risks, and costs.  b) Conditions for providing emergency care:  i) Technical-administrative capacity  ii) Financial and patrimonial proficiency  iii) Technological-scientific capacity |
| Triage classification | It defines technical criteria for the selection and classification of patients in emergency departments “Triage”. Specifically, 5 triage categories are described: Critical/Resuscitation, Emergency, Urgency, Minor Urgency, and Non-urgency. Besides, it outlines how triage systems can deployed in the wild. | Triage categories:  i) Critical/Resuscitation  ii) Emergency  iii) Urgency  iv) Minor Urgency  v) Non-urgency |
