# Supplementary material for: An integrated approach for designing in-time and economically sustainable emergency care networks: A case study in the public sector
Source: PLoS One. 2020 Jun 22;15(6):e0234984. doi: 10.1371/journal.pone.0234984 (PMC7307761; doi:10.1371/journal.pone.0234984)
Supplement: S2 Table — (DOCX) [file pone.0234984.s002.docx]

| ECN stakeholders | Expectations |
| --- | --- |
| Ministry of health and social protection, National council of healthcare, Healthcare control agencies | - Nimble attention in ECN - Low readmission risk - Respect and support from physicians and nurses - Accessibility - Efficient use of resources - Correct and complete provision of patients’ information |
| Social safety funds | - Nimble attention in ECN - Respect and support from physicians and nurses - Accessibility - Correct and complete provision of patients’ information |
| Healthcare promotion companies | - Nimble attention in ECN - Low readmission risk - Respect and support from physicians and nurses - Correct and complete provision of patients’ information |
| ECN Patients | - Nimble attention in ECN - EDs without overcrowding - Low transport times in ambulances - Availability of patient educational materials regarding triage classification - Appropriate diagnosis - Reasonable medical attention - Ward quality and privacy - Respect and support from physicians and nurses - Availability of drugs and supplies - Availability of appropriate and modern medical equipment - Safe care - Accessibility |
| Universities and other academic institutions | - Approval for executing research projects. - Employment of med students and trainees. |
| ED managers | - Nimble attention in ECN - Equitable distribution of payments - Low admission risk - Correct and complete provision of patients’ information |
| ECN Physicians, ECN nurses | - Eds without overcrowding - Appropriate ECN layout - Availability of drugs and supplies - Satisfactory working conditions - Availability of appropriate and modern medical equipment - Correct and complete provision of patient’s information |
| Hospitalization departments, intensive care units, surgery | - Correct and complete provision of patients’ information - Patient transferred considering protocols. |
| Laboratories | - Correct and complete provision of patients’ information - Optimal number of lab tests per patient - Correct and complete processing of lab test requests |
| Department of diagnostic imaging | - Correct and complete provision of patients’ information - Optimal number of diagnostic images per patient - Correct and complete processing of diagnostic imaging requests |
| Supplies and drug management | - Correct and complete provision of patients’ information - Optimal use of supplies and drugs. - Correct and complete processing of recipes and supply requests |
| Ambulance services | - Correct and complete provision of patients’ information - Timely provision of patient transfer information (including receiving node and protocols) |
