# Supplementary material for: An integrated approach for designing in-time and economically sustainable emergency care networks: A case study in the public sector
Source: PLoS One. 2020 Jun 22;15(6):e0234984. doi: 10.1371/journal.pone.0234984 (PMC7307761; doi:10.1371/journal.pone.0234984)
Supplement: S3 Table — (DOCX) [file pone.0234984.s003.docx]

| Potential failure mode | Potential failure effects | S | Potential causes | F | Current controls | D | RPN |
| --- | --- | --- | --- | --- | --- | --- | --- |
| Wrong triage classification | Higher mortality rate | 9 | Misjudgment of the physical symptoms | 5 | None | 10 | 450** |
|  | Longer ER length of stay | 5 | Misjudgment of the physical symptoms | 5 | None | 10 | 250* |
| Patient transfer delay | Development of more severe complications | 8 | Delayed authorization from HPCs | 5 | Delay analysis through indicators | 1 | 40 |
|  |  |  | Heavy traffic | 5 | Google Maps | 1 | 40 |
|  |  |  | Ambulance breakdown | 2 | Decision support system – Ambulance services | 1 | 16 |
|  | Higher mortality rate | 9 | Delayed authorization from HPCs | 5 | Delay analysis through indicators | 1 | 45 |
|  |  |  | Heavy traffic | 5 | Google Maps | 1 | 45 |
|  |  |  | Ambulance breakdown | 2 | Decision support system – Ambulance services | 1 | 18 |
|  | Longer ER length of stay | 5 | Delayed authorization from HPCs | 5 | Delay analysis through indicators | 1 | 25 |
|  |  |  | Heavy traffic | 5 | Google Maps | 1 | 25 |
|  |  |  | Ambulance breakdown | 2 | Decision support system – Ambulance services | 1 | 10 |
|  | Low patient satisfaction | 3 | Delayed authorization from HPCs | 5 | Delay analysis through indicators | 1 | 15 |
|  |  |  | Heavy traffic | 5 | Google Maps | 1 | 15 |
|  |  |  | Ambulance breakdown | 2 | Decision support system – Ambulance services | 1 | 6 |
| Delay to triage | Development of more severe complications | 8 | Shortage of nursing staff | 5 | Annual capability analysis | 6 | 240** |
|  |  |  | Shortage of medical staff | 5 | Annual capability analysis | 6 | 240** |
|  |  |  | Lack of triage rooms | 5 | Annual capability analysis | 6 | 240** |
|  |  |  | Delay during triage classification | 5 | None | 10 | 400** |
|  | Higher mortality rate | 9 | Shortage of nursing staff | 5 | Annual capability analysis | 6 | 270** |
|  |  |  | Shortage of medical staff | 5 | Annual capability analysis | 6 | 270** |
|  |  |  | Lack of triage rooms | 5 | Annual capability analysis | 6 | 270** |
|  |  |  | Delay during triage classification | 5 | None | 10 | 450** |
|  | Longer ER length of stay | 5 | Shortage of nursing staff | 5 | Annual capability analysis | 6 | 150* |
|  |  |  | Shortage of medical staff | 5 | Annual capability analysis | 6 | 150* |
|  |  |  | Lack of triage rooms | 5 | Annual capability analysis | 6 | 150* |
|  |  |  | Delay during triage classification | 5 | None | 10 | 250* |
|  | Low patient satisfaction | 3 | Shortage of nursing staff | 5 | Annual capability analysis | 6 | 90 |
|  |  |  | Shortage of medical staff | 5 | Annual capability analysis | 6 | 90 |
|  |  |  | Lack of triage rooms | 5 | Annual capability analysis | 6 | 90 |
|  |  |  | Delay during triage classification | 5 | None | 10 | 150* |
| No access to entrance | Development of more severe complications | 8 | Overcrowding | 9 | Decision support systems/Delay analysis through indicators | 1 | 72 |
|  | Higher mortality rate | 9 | Overcrowding | 9 | Decision support systems/Delay analysis through indicators | 1 | 81 |
|  | Longer ER length of stay | 5 | Overcrowding | 9 | Decision support systems/Delay analysis through indicators | 1 | 45 |
|  | Low patient satisfaction | 3 | Overcrowding | 9 | Decision support systems/Delay analysis through indicators | 1 | 27 |
| Delay to quick register | Development of more severe complications | 8 | Shortage of receptionists | 2 | Annual capability analysis | 6 | 96 |
|  |  |  | Unavailable user information system | 3 | Maintenance Inspection and reports of failures | 1 | 24 |
|  |  |  | Extended patient admission process | 2 | None | 10 | 160** |
|  | Higher mortality rate | 9 | Shortage of receptionists | 2 | Annual capability analysis | 6 | 108 |
|  |  |  | Lack of an user information system | 3 | Maintenance Inspection and reports of failures | 1 | 27 |
|  |  |  | Extended patient admission process | 2 | None | 10 | 180** |
|  | Longer ER length of stay | 5 | Shortage of receptionists | 2 | Annual capability analysis | 6 | 60 |
|  |  |  | Lack of an user information system | 3 | Maintenance Inspection and reports of failures | 1 | 15 |
|  |  |  | Extended patient admission process | 2 | None | 10 | 100 |
|  | Low patient satisfaction | 3 | Shortage of receptionists | 2 | Annual capability analysis | 6 | 36 |
|  |  |  | Lack of an user information system | 3 | Maintenance Inspection and reports of failures | 1 | 9 |
|  |  |  | Extended patient admission process | 2 | None | 10 | 60 |
| No ambulance available | Development of more severe complications | 8 | Shortage of ambulances | 5 | Decision support system – Ambulance services | 1 | 40 |
|  |  |  | Ambulance breakdown | 2 | Decision support system – Ambulance services | 1 | 16 |
|  | Higher mortality rate | 9 | Shortage of ambulances | 5 | Decision support system – Ambulance services | 1 | 45 |
|  |  |  | Ambulance breakdown | 2 | Decision support system – Ambulance services | 1 | 18 |
|  | Longer ER length of stay | 5 | Shortage of ambulances | 5 | Decision support system – Ambulance services | 1 | 25 |
|  |  |  | Ambulance breakdown | 2 | Decision support system – Ambulance services | 1 | 10 |
