# Supplementary material for: An integrated approach for designing in-time and economically sustainable emergency care networks: A case study in the public sector
Source: PLoS One. 2020 Jun 22;15(6):e0234984. doi: 10.1371/journal.pone.0234984 (PMC7307761; doi:10.1371/journal.pone.0234984)
Supplement: S4 Table — (DOCX) [file pone.0234984.s004.docx]

| Potential failure mode | Potential failure effect | Potential cause | Recommended actions |
| --- | --- | --- | --- |
| Wrong triage classification | Higher mortality rate | Misjudgment of the physical symptoms | -Train triage doctors to classify patients correctly.  - Establish a p control chart for the proportion of wrong-triaged patients. |
| Wrong triage classification | Longer ER length of stay | Misjudgment of the physical symptoms |  |
| Delay to triage | Development of more severe complications | Shortage of nursing staff | -Perform short-term load analysis (every month) to determine the required nursing and medical staff in triage.  -According to the previous point, hire the required doctors and nurses (if necessary) |
| Delay to triage | Development of more severe complications | Shortage of medical staff |  |
| Delay to triage | Development of more severe complications | Lack of triage rooms | -Implementation of fast-track triage. |
| Delay to triage | Development of more severe complications | Delay during triage classification | **-** Perform a VSM analysis to detect and eliminate non-value activities during triage classification. |
| Delay to triage | Higher mortality rate | Shortage of nursing staff | -Perform short-term load analysis (every month) to determine the required nursing and medical staff in triage.  -According to the previous point, hire the required doctors and nurses (if necessary) |
| Delay to triage | Higher mortality rate | Shortage of medical staff |  |
| Delay to triage | Higher mortality rate | Lack of triage rooms | -Implementation of fast-track triage. |
| Delay to triage | Higher mortality rate | Delay during triage classification | **-** Perform a VSM analysis to detect and eliminate non-value activities during triage classification. |
| Delay to triage | Longer ER length of stay | Shortage of nursing staff | -Perform short-term load analysis (every month) to determine the required nursing and medical staff in triage.  -According to the previous point, hire the required doctors and nurses (if necessary) |
| Delay to triage | Longer ER length of stay | Shortage of medical staff |  |
| Delay to triage | Longer ER length of stay | Lack of triage rooms | -Implementation of fast-track triage. |
| Delay to triage | Longer ER length of stay | Delay during triage classification | **-** Perform a VSM analysis to detect and eliminate non-value activities during triage classification. |
| Delay to triage | Low patient satisfaction | Delay during triage classification |  |
| Delay to quick register | Development of more severe complications | Extended patient admission process | **-** Perform a VSM analysis to detect and eliminate non-value activities during admission process. |
| Delay to quick register | Higher mortality rate | Extended patient admission process |  |
