# Supplementary material for: An integrated approach for designing in-time and economically sustainable emergency care networks: A case study in the public sector
Source: PLoS One. 2020 Jun 22;15(6):e0234984. doi: 10.1371/journal.pone.0234984 (PMC7307761; doi:10.1371/journal.pone.0234984)
Supplement: S5 Table — (DOCX) [file pone.0234984.s005.docx]

| Process variable | | Expression | P-value |
| --- | --- | --- | --- |
| Time between  arrivals (min) | M-P1 | 514 * BETA(0.917, 5.4) | 0.0883 |
|  | M-P2 | EXPO(24.5) | 0.184 |
|  | M-P3 | EXPO(21.3) | >0.75 |
|  | Tu-P1 | -0.001 + WEIB(63.9, 1.06) | 0.282 |
|  | Tu-P2 | WEIB(22.5, 1.07) | 0.466 |
|  | Tu-P3 | EXPO(18.5) | 0.554 |
|  | W-P1 | EXPO(57.2) | 0.034 |
|  | W-P2 | EXPO(23.8) | 0.26 |
|  | W-P3 | EXPO(19.5) | 0.707 |
|  | Th-P1 | GAMM(59.7, 1) | 0.75 |
|  | Th-P2 | GAMM(23.9, 0.942) | >0.75 |
|  | Th-P3 | EXPO(20) | 0.508 |
|  | F-P1 | GAMM(57.1, 1.01) | 0.75 |
|  | F-P2 | GAMM(22, 0.991) | >0.75 |
|  | F-P3 | EXPO(19.4) | 0.168 |
|  | Sa-P1 | GAMM(51.9, 1.05) | >0.75 |
|  | Sa-P2 | GAMM(20, 0.989) | >0.75 |
|  | Sa-P3 | GAMM(14.5, 1.07) | 0.653 |
|  | Su-P1 | EXPO(52.4) | 0.341 |
|  | Su-P2 | GAMM(20.1, 1.01) | 0.75 |
|  | Su-P3 | EXPO(17.4) | 0.598 |
| Triage time per patient (min) | | UNIF (2, 5) | >0.15 |
| Admission time (min) | | UNIF (5, 10) | >0.15 |
| Bed preparation time (min) | | UNIF (5, 10) | >0.15 |
| Nursing assistance time (min) | | UNIF (6, 7) | >0.15 |
| Physician assessment time (min) | | UNIF (5, 27) | 0.072 |
| Treatment time (min) | | 19 + WEIB (247, 1.21) | 0.718 |
